# RASSF1A uncouples Wnt from Hippo signalling and promotes YAP mediated differentiation via p73

**DOI:** 10.1038/s41467-017-02786-5

**Published:** 2018-01-30

**Authors:** Angelos Papaspyropoulos, Leanne Bradley, Asmita Thapa, Chuen Yan Leung, Konstantinos Toskas, Delia Koennig, Dafni-Eleftheria Pefani, Cinzia Raso, Claudia Grou, Garth Hamilton, Nikola Vlahov, Anna Grawenda, Syed Haider, Jagat Chauhan, Ludovico Buti, Alexander Kanapin, Xin Lu, Francesca Buffa, Grigory Dianov, Alex von Kriegsheim, David Matallanas, Anastasia Samsonova, Magdalena Zernicka-Goetz, Eric O’Neill

**Affiliations:** 1 CRUK/MRC Oxford Institute, Department of Oncology, University of Oxford, Oxford, OX3 7DQ UK; 2 The Wellcome Trust/Cancer Research UK Gurdon Institute, University of Cambridge, Cambridge, CB2 1QN UK; 3 Department of Physiology, University of Cambridge, Cambridge, CB2 3EG UK; 4 Systems Biology Ireland, University College Dublin, Dublin 4, Ireland; 5 Applied Computational Genomics, Department of Oncology, University of Oxford, Oxford, OX3 7DQ UK; 6 Ludwig Institute for Cancer Research, Nuffield Department of Clinical Medicine, University of Oxford, Oxford, OX3 7DQ UK; 7 Bioinformatics Research Core, Department of Oncology, University of Oxford, Oxford, OX3 7DQ UK; 8 Institute of Cytology and Genetics, Russian Academy of Sciences, Lavrentyeva 10, Novosibirsk, 630090 Russian Federation

## Abstract

Transition from pluripotency to differentiation is a pivotal yet poorly understood developmental step. Here, we show that the tumour suppressor RASSF1A is a key player driving the early specification of cell fate. RASSF1A acts as a natural barrier to stem cell self-renewal and iPS cell generation, by switching YAP from an integral component in the β-catenin-TCF pluripotency network to a key factor that promotes differentiation. We demonstrate that epigenetic regulation of the *Rassf1A* promoter maintains stemness by allowing a quaternary association of YAP–TEAD and β-catenin–TCF3 complexes on the *Oct4* distal enhancer. However, during differentiation, promoter demethylation allows GATA1-mediated RASSF1A expression which prevents YAP from contributing to the TEAD/β-catenin–TCF3 complex. Simultaneously, we find that RASSF1A promotes a YAP–p73 transcriptional programme that enables differentiation. Together, our findings demonstrate that RASSF1A mediates transcription factor selection of YAP in stem cells, thereby acting as a functional “switch” between pluripotency and initiation of differentiation.

## Abstract (web summary)

In development, the switch from pluripotency to differentiation is important but it is often unclear how it is regulated. Here, the authors show that the tumour suppressor RASSF1A mediates this switch by promoting YAP-p73 transcription, which in turn enables differentiation.

## Introduction

WNT, Notch, Hedgehog and Hippo signalling pathways play important roles in maintaining pluripotency^1^. The transcriptional co-factors of the Hippo pathway, YAP and TAZ (Wwtr1) promote stem cell self-renewal and pluripotency by mediating TEA-domain (TEAD1-4) transcription. Of the TEA domain family of transcription factors, TEAD2 is upregulated in embryonic stem cells (ESC)^2^ with TEAD2 and TEAD4 expressed early in embryogenesis^3, 4^. YAP–TEAD transcriptional complexes directly activate *Pou5f1* (Oct4; hereafter *Oct4*) expression and the subsequent expansion of pluripotent cells^5^. YAP plays a crucial role in cell specification of developing embryonic blastocysts, where in line with studies in ESCs, YAP is also nuclear and drives pluripotency in the inner cell mass (ICM)^6^. However, definition of the trophectoderm (TE) depends on YAP-mediated expression of CDX2^4^. This dichotomous role of YAP in the blastocyst has been suggested to be due to transcriptional cooperation with β-catenin in the ICM, but also suggests that YAP may have distinct roles in pluripotent versus differentiating cells, such as the TE. Accumulating evidence supports roles for YAP and TAZ in early ESC differentiation and terminal differentiation of tissues, guided by distinct transcription factor selection^7–10^. The WNT pathway effector TCF1/LEF can also actively repress or activate expression of *Oct4*, depending on β-catenin association. In this manner, β-catenin plays a major role as a mediator of WNT signaling in maintaining stem cell self-renewal^11, 12^ but requires YAP to drive dedifferentiation of cardiac progenitors^13^, indicating that WNT and Hippo converge to regulate pluripotency.

The Hippo pathway is a signal transduction cascade where the MST1/2 kinases activate LATS/NDR kinases to phosphorylate YAP/TAZ. SAV1 and RASSF1-6 are direct binding scaffolds of MST1/2 that regulate and localise pathway activation and YAP activity. LATS/NDR-mediated phosphorylation of YAP on five serine residues prevents association with TEADs and the pSer127-YAP (Ser112 in mouse) site presents a binding site for 14-3-3 proteins that increases cytoplasmic retention^14^. During differentiation of ESCs, activation of Hippo signalling restricts TEAD-mediated transcription of *Oct4*, however, exactly how the Hippo pathway is activated during differentiation is not known^5^.

While the pathways regulating pluripotency are well understood, those driving early stages of transition to a differentiated state remain elusive. The earliest specification of stem cell fate upon exiting pluripotency has recently been reported to be dependent on p73^15^, a transcriptional partner of the Hippo pathway mediator YAP, that is also required during terminal differentiation^16–18^. Activation of the Hippo pathway, resulting in increased pSer127-YAP, is also required for optimal association with p73^19–22^. Moreover, RASSF1A-mediated Hippo pathway activation promotes both pS127-YAP and the formation of active YAP–p73 transcriptional complexes, while loss of RASSF1A expression allows the formation of pro-proliferative YAP–TEAD^21, 23, 24^. *RASSF1A* silencing via promoter hypermethylation is widespread in solid malignancies and correlates with a loss of pS127-YAP, elevated YAP/β−catenin mediated transcription and increased ESC signatures in tumors^25–27^. Furthermore, human mesenchymal stem cells harbouring targeted promoter methylation of *RASSF1A* display oncogenic properties and maintain stemness^28^. Thus, methylation of *RASSF1A* is proposed to induce dedifferentiation of tumour cells, which may account for poor prognosis of patients whose cancers are associated with high levels of epigenetic silencing^29, 30^.

A potential role for RASSF1A in early development may be indicated as methylation of the *Rassf1A* promoter is observed in both mouse and human oocytes/zygotes and decreases as cells pass through 2–8 cell stages of the pre-implantation embryo, in line with increased mRNA expression^31^. These events occur inversely to the methylation of *Nanog* and *Oct4* during the epigenetic reprogramming of the embryo and point to a more sophisticated regulation on the epigenome during embryogenesis. As frequently observed for regulatory components of the pre-implantation embryo and most recently demonstrated for p73^4, 32^, genetic ablation of individual Hippo components (e.g., *Mst1*, *Mst2*, *Rassf1*) in mice can demonstrate mild or late phenotypes due to redundancy and compensation. Importantly in humans, epigenetic silencing of *RASSF1A* expression is associated with developmental defects in the placenta, pointing to an underappreciated role in early development^33^.

Here, we provide evidence that RASSF1A regulation of the Hippo pathway effector YAP controls the balance between pluripotency and differentiation of ESCs. We provide evidence that YAP–TEAD2 and β-catenin–TCF3 cooperate on the *Oct4* promoter to promote expression and pluripotency under stem cell maintenance conditions. Differentiation induces expression of RASSF1A, removing YAP from the TEAD2 complex and disabling *Oct4* transcription. We find that RASSF1A-induced pS127-YAP is not inactive but plays an active role in differentiation through direct binding to p73 and activation of lineage specification genes.

## Results

### RASSF1A is expressed in differentiating stem cells

**Fig. 1:**
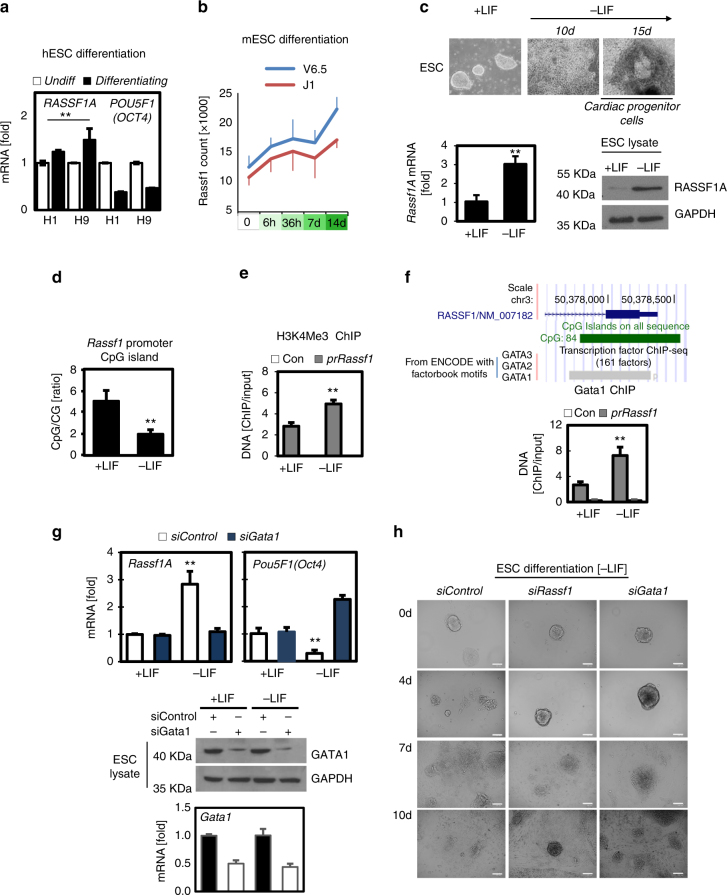
Epigenetic activation of *Rassf1A* drives ESC differentiation via GATA1. **a** *RASSF1A* and *Pou5f1/Oct4* mRNA levels in undifferentiated and differentiating human ESC lines H1 and H9. Data obtained from published GSE54186 data set. **b** *Rassf1A* mRNA levels increase upon LIF withdrawal-mediated differentiation of mouse ESC (mESC) lines V6.5 and J1. Data obtained from published GSE3749 and GDS2672 data sets. **c** *Rassf1A* mRNA (bars) and protein (blots) levels from undifferentiated (+LIF) versus differentiated (-LIF) E14Tg2a mESC. See also Supplementary Fig. 1b and Supplementary Movie 1. **d** *Rassf1A* CpG island promoter methylation (assessed by qPCR for methylated/unmethylated DNA ratio) and **e** H3K4me3 levels in pluripotent (+LIF) versus differentiated (-LIF) mESC. See also Supplementary Fig. 1a. **f** ENOCDE transcription factor ChIP data showing specific binding of GATA1 on the *Rassf1A* promoter of differentiated hESC and GATA1 ChIP on the *Rassf1A* promoter of pluripotent (+LIF) versus differentiated (-LIF) ESC. **g** qPCR for *Rassf1A* and *Oct4* mRNA levels in response to siRNA-mediated *Gata1* silencing in the presence or absence of LIF in ESC. Western blotting and qPCR indicate the level of Gata1 KD. See also Supplementary Figs 1d–e, 2c and 5d. **h** Representative ESC colonies from indicated conditions subject to LIF withdrawal assay. Scale bars: 25–50 μm. **P < 0.01 of Student’s *t*-test. Error bars indicate s.e.m. Data shown are representative of at least three independent experiments

Epigenetic regulation of genes has been widely implicated in stem cell fate specification^34^. *RASSF1A* expression is widely observed to be epigenetically regulated in cancer, and demethylation of the human *RASSF1A* promoter has been shown to accompany differentiation of neural progenitors into somatic cells^35^. To determine whether RASSF1A played a role in stem cell differentiation we first examined levels of *RASSF1A* in human (H1 and H9) and mouse (J1 and V6.5) ESC lines allowed to differentiate over a period of 7–14 days. h*RASSF1A* and m*Rassf1A* (hereafter *Rassf1A*) mRNA expression increased upon differentiation of both human and mouse ESCs (Fig. 1a, b). Using the ENCODE Project database to compare human ESC and mesoderm-derived progenitors, we find the *RASSF1* promoter appears in a ‘poised’ epigenetic state in ESCs rather than completely silenced, as the promoter is marked with both activating H3K4^me2^ and repressive H3K27^me3^ histone modifications (Supplementary Fig. 1a). Differentiation into mesoderm progenitors is coincident with loss of the repressive mark specifically at the *RASSF1* promoter CpG island, conversion of H3K4^me2^ to H3K4^me3^ (a mark of transcriptional activity) and increased transcription (Supplementary Fig. 1a). To confirm changes in endogenous expression, a pool of E14Tg2a mESC were allowed to terminally differentiate in the absence of LIF (-LIF), indicated from the existence of beating cardiac progenitor cells (Fig. 1c, Supplementary Fig. 1b and Supplementary Movie 1). Levels of both endogenous *Rassf1A* mRNA and protein were elevated in differentiated (-LIF) cells compared to the undifferentiated (+LIF) cells (Fig. 1c). Moreover, we find reduced CpG island promoter hypermethylation and increased active transcription H3K4^me3^ chromatin marks at the *RASSF1A* promoter in differentiated versus pluripotent mouse ESCs (Fig. 1d, e and Supplementary Fig. 1a). Thus, demethylation of *Rassf1A* along with elevated mRNA expression occurs when cells move from a pluripotent state towards a more terminally differentiated fate at the same time as expression of pluripotency genes *Nanog* and *Oct4* are silenced through increased DNA methylation.

Analysis of the *Rassf1A* promoter for consensus binding sites (TRANSFAC), reveals direct evidence (ChIP, ENCODE) only for binding by the differentiation-promoting GATA family member^36^, GATA1 (Fig. 1f). GATA transcription factors regulate haematopoietic lineage commitment and have been increasingly described to have roles in cell differentiation in intestinal crypts and during early embryogenesis^37, 38^. We found selective binding of GATA1 to the *Rassf1A* promoter and a positive association with *Rassf1A* transcription in differentiating cells (Fig. 1f and Supplementary Fig. 1c). *Gata1* silencing was sufficient to minimise *Rassf1A* expression in the absence of LIF, while ectopic expression of GATA1 induced expression despite the presence of LIF (Fig. 1g, Supplementary Fig. 1d). Furthermore, reduction of *Gata1* mRNA resulted in maintenance of *Oct4* expression in the absence of LIF, indicating that ESCs fail to differentiate in the absence of GATA1-driven RASSF1A expression (Fig. 1g, Supplementary Fig. 1d). Taken together, these data indicate that epigenetic changes on the *Rassf1A* promoter are permissive for GATA1 binding which in turn supports *Rassf1A* expression upon differentiation (Supplementary Fig. 1e). Loss of either *Gata1* or *Rassf1A* expression resulted in ESC colonies failing to appropriately differentiate and persisting as pluripotent-like colonies in the absence of LIF (Fig. 1h).

### RASSF1A regulates expression of differentiation genes

**Fig. 2:**
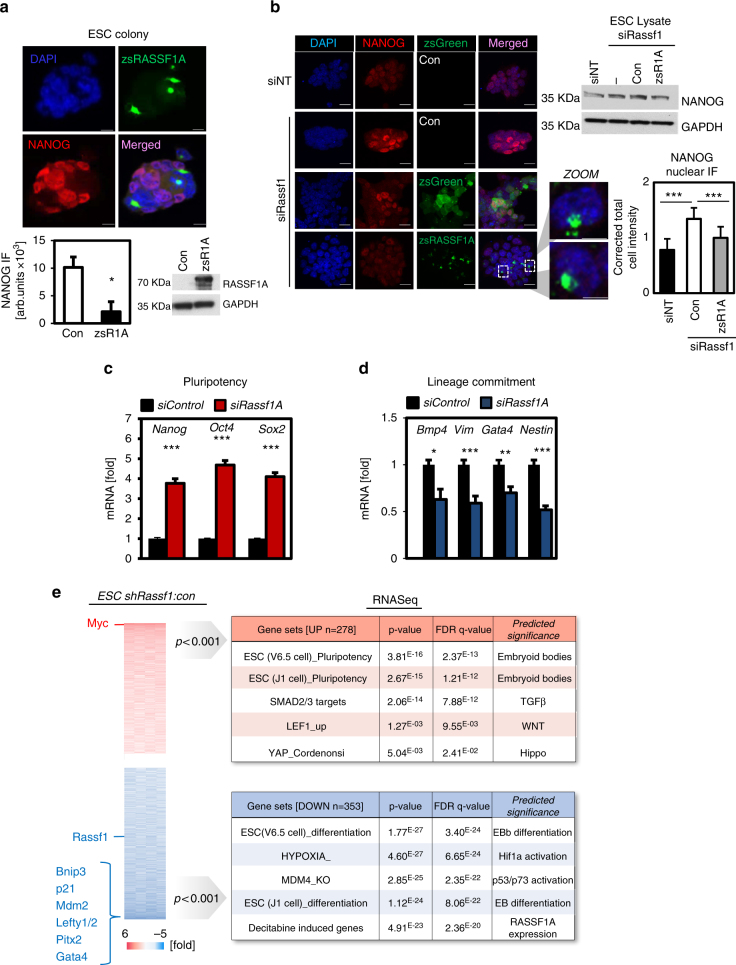
RASSF1A regulates the ESC core pluripotency network. **a** Representative fluorescent images of Nanog and zsRASSF1A (zsR1A)-expressing cells in mouse ESC colonies. Bar graph and western blotting represent quantification of Nanog and RASSF1A levels, respectively. **b** Nanog immunofluorescence in siNT versus siRASSF1A-transfected ESC. Additional expression of siRASSF1A-resistant zsRASSF1A rescues the phenotype in siRASSF1A-transfected ESC, quantified in the displayed bar graphs and additionally demonstrated by Western blotting. Zoom in displays RASSF1A localisation peripherally to the nucleus at the microtubule organising centre, in zsRASSF1A-expressing cells. Validation using a second siRNA to *Rassf1A* is provided in Supplementary Fig. 2g. **c** qPCR for core stem cell markers from ESC in **b**. Ectopic expression of RASSF1A reverses the ESC pluripotent phenotype, see Supplementary Fig 2a–e. **d** qPCR for germ layer-specific differentiation markers in ESC subject to LIF withdrawal. **e** RNAseq analysis in shRNA-expressing ESC versus control (shGFP) reveals establishment of self-renewal and pluripotency signatures in the absence of *Rassf1A*, Supplementary Data 1. Scale bars: 25 and 50 μm. *P < 0.05, **P < 0.01 and ***P < 0.001, respectively, of Student’s *t*-test. Error bars indicate s.e.m. Data shown are representative of at least three independent experiments

To test whether increased levels of RASSF1A plays an active role in differentiation, we first transiently expressed RASSF1A in mESC maintained under pluripotent conditions. RASSF1A-positive cells were less positive for the stem cell marker NANOG (Fig. 2a and Supplementary Fig. 2a, c, e), had reduced number of ESC colonies and reduced expression of the pluripotency gene cassette (*Nanog, Oct4, Sox2*) (Supplementary Fig. 2b, d). Conversely, ESC depleted of *Rassf1A*, had enhanced levels of NANOG expression which could be rescued by ectopic expression of zsRASSF1A, but not by a derivative incapable of activating MST kinases due to deletion of the C-terminus (Fig. 2b and c and Supplementary Fig. 2c). Additionally, ESC depleted of *Rassf1A* displaying increased *Nanog, Oct4, Sox2* demonstrated a reciprocal decrease in the expression of genes involved in lineage commitment and had increased pluripotent-like ESC colonies (Fig. 2d and Supplementary Fig. d, f). To determine whether ESC potency was directly regulated by RASSF1A, we performed RNA sequencing in shRassf1A-expressing ESC versus control (shGFP) (Fig. 2e). In the absence of RASSF1A, genes required to maintain pluripotency and self-renewal were upregulated, including components of the TGFβ, WNT and Hippo pathways, SMAD2/3, LEF1 and YAP targets, whereas factors promoting ESC differentiation, such as p53/p73 targets, *Cdkn1A* (p21) and *Gata4*, were downregulated (Fig. 2e and Supplementary Data 1). Expression of RASSF1A is therefore sufficient to directly regulate expression of pluripotency versus differentiation genes.

### A quaternary YAP complex induces *Oct4* expression

**Fig. 3:**
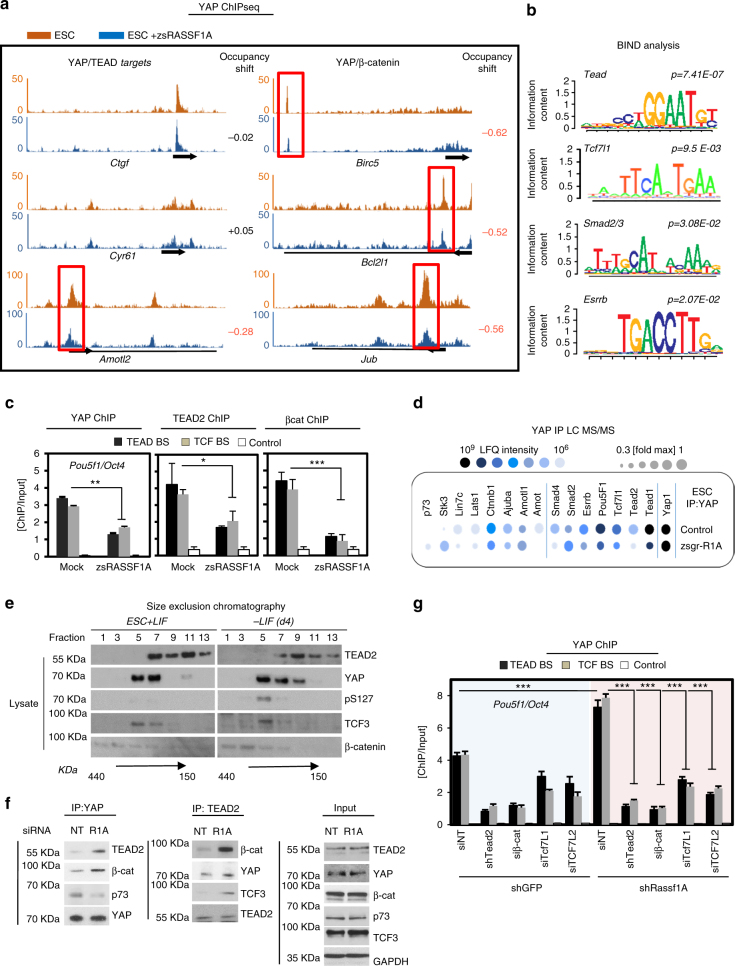
A quaternary YAP–TEAD2/β-catenin-TCF complex is essential for maintaining expression of *Oct4*. **a** YAP ChIP-seq in zsRASSF1A-overexpressing versus control ESC depicting reduced occupancy of YAP–TEAD and YAP-β-catenin target genes in the presence of RASSF1A. See also Supplementary Fig. 3a, b, 4 and Data 6. **b** Enriched sequence motifs (*P < 0.05*) identified in the proximity of upregulated YAP target genes upon RASSF1A loss in ESC. See also Supplementary Data 2. **c** YAP, TEAD2 and β-catenin ChIP on TEAD2 and TCF binding sites (BS) on the ESC *Pou5f1/Oct4* promoter in response to indicated conditions. **d** Proteomics analysis for YAP binding partners in empty vector versus zsRASSF1A-transfected ESC. Factors modifying their affinity with YAP are represented by relative abundance of peptides (LFQ intensity) and fold difference to maximum. All mass-spec intensities were normalised to YAP intensities. See also Supplementary Fig. 3c and Data 3. **e** Size exclusion chromatography of ESC lysates via Gel filtration column and western blotting with indicated antibodies. See also Supplementary Fig. 3d. **f** Western blotting of YAP and TEAD2 immunoprecipitates in response to transient depletion of RASSF1A. Validation using #2 siRassf1A and shRassf1A is provided in Supplementary Fig. 3e–g. **g** YAP ChIP at TEAD and β-catenin/TCF binding sites (BS) on the ESC *Pou5f1/Oct4* promoter in response to stable ablation of RASSF1A versus control. *P < 0.05, **P < 0.01 and ***P < 0.001, respectively, of Student’s *t*-test. Error bars indicate s.e.m. Data shown are representative of at least three independent experiments

RASSF1A is an established component of the Hippo pathway; we therefore set out to investigate whether its activation affects YAP–TEAD and YAP-β-catenin transcriptional complex formation/activity, which both have a pivotal role in maintaining stem cell self-renewal^2, 5, 13^. YAP ChIP-seq demonstrated reduced association (occupancy shift) of YAP on target genes in ESC expressing RASSF1A compared to controls. Previously documented YAP–TEAD/β−catenin target genes appeared more sensitive to RASSF1A levels than classical YAP–TEAD targets which displayed equivalent peaks in RASSF1A-expressing ESC and controls (Fig. 3a and Supplementary Fig. 3b). To assess the transcriptional activity of YAP in response to RASSF1A expression, we combined genome-wide profiling of YAP binding (ChIPseq) with transcriptome analysis (RNAseq) in shGFP and shRassf1A-expressing ESC lines. There was a substantial enrichment of YAP binding events in the immediate vicinity of transcription start sites of genes differentially expressed following knockdown of *Rassf1A*, indicating the major transcriptional activity of YAP is negatively regulated by RASSF1A in ESC (Supplementary Fig. 3a). As expected, TEAD was the most upregulated transcription factor motif at sites proximal to YAP targets in the absence of RASSF1A (Fig. 3b). Additionally, motifs of several pluripotency-related transcription factors including TCF7L1/2(TCF3/4), ESRRB, and SMAD2/3^39^ (Fig. 3b and Supplementary Data 2) were increased in the absence of RASSF1A. TEAD2, TCF3/4 and β-catenin activate *Oct4* transcription via direct binding of its distal enhancer^2, 40^. We therefore reasoned that RASSF1A may redirect YAP from active complexes on the *Oct4* promoter, preventing expression. Indeed, direct ChIP-qPCR for YAP, TEAD2 and β-catenin demonstrated that in the presence of RASSF1A; YAP, TEAD2 and β-catenin have decreased affinity for the *Oct4* promoter (Fig. 3c). In keeping with our previous results, analysis of the YAP interactome in ESCs revealed that TEAD2, TCF3 and its co-factor β-catenin^41^ lose association with YAP in response to RASSF1A expression (Fig. 3d, Supplementary Fig. 3c and Data 3). We next examined our separation of ESC lysates on gel filtration column and observed co-purification of total YAP in the same fraction as β-catenin, TCF3 and TEAD2 in the presence of LIF, however, the co-purification is lost upon differentiation (-LIF day 4; Fig. 3e and Supplementary Fig. 3d).

To confirm these observations, we depleted *Rassf1A* from ESCs either transiently using two independent siRNA sequences or stably, using lentiviral vectors and determined interaction by co-immunoprecipitation. Loss of *Rassf1A* resulted in elevated levels of β-catenin and TCF3 in YAP and TEAD2 immunoprecipitates (Fig. 3f, Supplementary Fig. 3e, f, g and 4a). The identification of TCF3 in YAP IPs suggests the existence of a YAP–TEAD2–β-catenin trimeric complex, which may include the canonical β-catenin partners TCF3/4. To address this, we employed stable shGFP, shRassf1A, shTead2 and shRassf1A/shTead2 ESC lines transfected with siRNAs to either β-catenin, TCF3 and TCF4, or non-targeting controls (siNT) (Supplementary Fig. 4b and c) and performed YAP ChIP using primers identifying either TEAD or β-catenin/TCF binding sites on the *Oct4* promoter. Knockdown of shRassf1A led to an increase in YAP occupancy of both sites on the *Oct4* promoter which was dependent on β-catenin, TCF3 and TCF4 (Fig. 3g). This implies that β-catenin localisation to the *Oct4* promoter is TEAD2-dependent and suggests that the YAP–TEAD2–β-catenin–TCF3 complex acts as transcription-promoting unit on *Oct4*. In support of this, silencing of β-catenin, TCF3 or TCF4 together with RASSF1A (shRassf1A/siβcat; shRassf1A/siTcf7l1; shRassf1A/siTcf7l2), reduced TEAD2 promoter binding (Fig. 3g). YAP depletion disrupted the complex formation, confirming that RASSF1A-mediated regulation of TEAD2-β-catenin was indeed YAP-dependent and responsible for *Oct4/Sox2/Nanog* expression and pluripotency (Supplementary Fig. 3g). Moreover, we performed TEAD2 and β-catenin ChIP on the same target sites of the *Oct4* promoter and obtained similar results (Supplementary Fig. 5a and b). In line with the transcriptional regulation of *Rassf1A*, GATA1 overexpression also suppresses the ability of YAP to ChIP at both binding sites in the *Oct4* enhancer (Supplementary Fig. 5c). Intriguingly, in addition to silencing of YAP, TEAD2 or β-catenin (Supplementary Fig. 4d–f), silencing of MST2-LATS1 in the absence of RASSF1A ablated *Oct4* and *Sox2* upregulation (Supplementary Fig. 5d). This suggests that the RASSF1A signalling switch of the YAP partner not only operates through the Hippo cassette, but may be required to facilitate YAP-β−catenin transcription over YAP–TEAD alone as suggested previously^26^.

### RASSF1A promotes YAP and p73 binding to differentiation genes

**Fig. 4:**
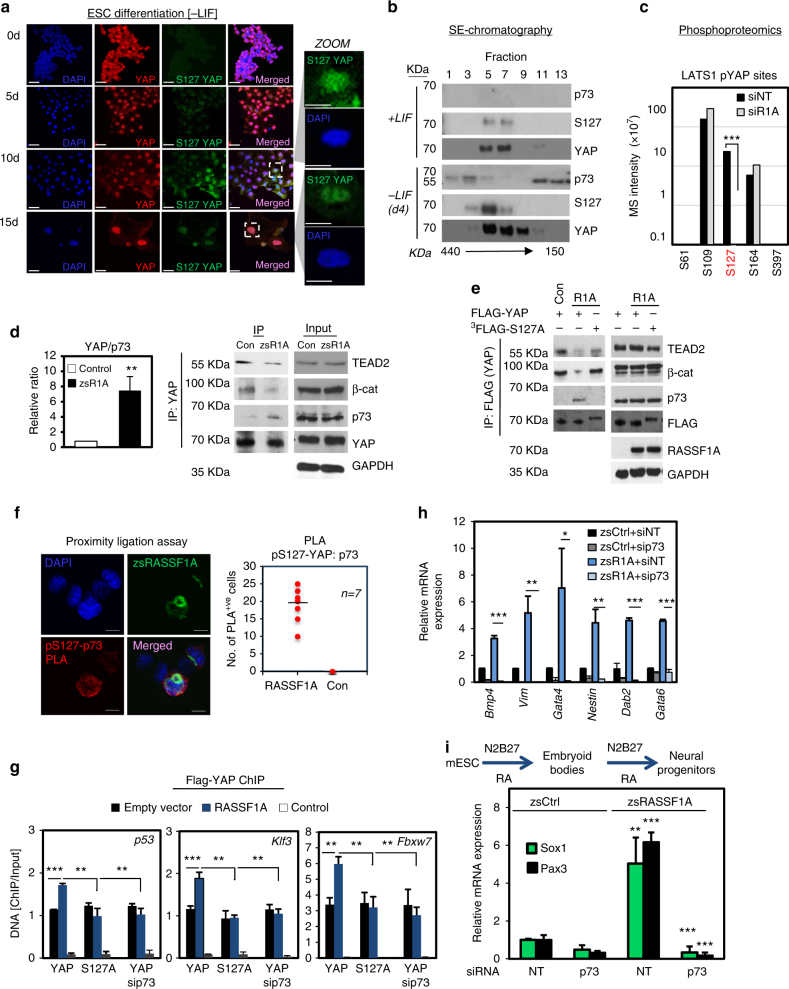
RASSF1A regulates LATS-mediated YAP phosphorylation and enforces differentiation entry via p73. **a** Subcellular localisation and expression of total YAP and pS127-YAP in gradually differentiating ESC cultures. pS127-YAP is significantly increased upon differentiation. **b** Size exclusion chromatography of ESC lysates via Gel filtration column and western blotting with indicated antibodies. **c** Phosphoproteomic analysis of YAP phosphorylation in RASSF1A-expressing versus non-expressing ESC. All cells are transfected with human FLAG–YAP1. All mass-spec intensities were normalised to YAP intensities for each sample. See also Supplementary Fig 6a and Data 4. **d** Western blotting of YAP immunoprecipitates under indicated conditions for the indicated antibodies and quantification of p73 relative ratio to YAP. **e** Western blotting of FLAG immunoprecipitates from ESC transfected with the indicated constructs. FLAG–YAP mutants are triple-tagged. **f** Proximity ligation assay (PLA) demonstrates association of anti-pS127-YAP with anti-p73 antibodies. Red dots indicate positive association. The graph reports number of PLA events between p73/pS127 in the presence or absence of RASSF1A. See also Supplementary Fig 6b. **g** YAP ChIP on indicated differentiation-related gene promoters and **h** qPCR for germ layer-specific differentiation markers in ESC in response to indicated conditions. See also Supplementary Data 6. **i** ESC cultures expressing zsRASSF1A or empty vector (zsCtrl) were subject to neural differentiation assay via N2B27 and retinoic acid (RA). Scale bars: 25–50 μm. *P < 0.05, **P < 0.01 and ***P < 0.001, respectively, of Student’s *t*-test. Error bars indicate s.e.m. Data shown are representative of at least three independent experiments

LATS-dependent phosphorylation of YAP regulates its activity in response to various signals^42, 43^. As shown previously, the key LATS-dependent regulatory site, pS127-YAP, is undetectable in pluripotent ESCs and increases during ESC differentiation^5^. We also find this is the case, however, rather than purely cytoplasmic a proportion of pS127-YAP remains nuclear at day 5 –LIF and becomes largely nuclear by day 15 (Fig. 4a). We also reasoned that since RASSF1A drives the formation of pro-apoptotic YAP–p73 complexes in cancer^21, 23, 24^, expression of RASSF1A may also stabilise YAP–p73 complexes in ESCs and promote differentiation. We next examined our Gel filtration separation of ESC d4 –LIF lysates and observed co-purification of a small pool of total YAP marked with pS127-YAP in the same high molecular weight fraction as p73 (Fig. 4b), indicating the potential for complex formation. We performed a phosphoproteomic analysis of FLAG–YAP immunoprecipitates from ESCs in the presence or absence of RASSF1A and, in line with a role for the Hippo pathway, S127-YAP was phosphorylated when RASSF1A was expressed (Fig. 4c, Supplementary Fig. 6a and Data 4). Moreover, in pluripotent ESCs, YAP–TEAD–β-catenin–ΤCF3 complexes were readily detected, but were reduced in favour of YAP–p73 in the presence of RASSF1A (Fig. 3e, f and Supplementary Data 3). To investigate whether YAP–p73 complexes play a role in ESC fate determination as described for p73^15^, we examined genome-wide YAP DNA binding peaks (ChIPseq). This showed that RASSF1A enriched YAP binding to several differentiation gene promoters (e.g., *p53*, *Fbxw7, Klf3*) containing p73 binding consensus motifs^44, 45^ (Supplementary Data 5). p53/p73 can promote BMP4 expression during differentiation^46^ and Fbxw7 is an E3 ligase that targets c-MYC, which is linked with development of endothelial and hematopoietic lineages in the embryo^47^ and regulates GATA4^48^.

### YAP and p73 complex stabilisation by RASSF1A is dependent on pS127

As RASSF1A signaling stabilises YAP–p73 complexes (Fig. 4d), we speculated that the formation of these complexes may be dependent on phosphorylation of S127. To directly address this, ESCs were transiently transfected with either FLAG–YAP or a non-phosphorylatable^3^ FLAG–YAP-S127A mutant and complexes were retrieved by co-immunoprecipitation of the FLAG epitope. RASSF1A expression promoted the switch of YAP association with TEAD2 and β-catenin to p73, however, in the context of the S127A mutant TEAD2 and β-catenin binding was maintained (Fig. 4e). Furthermore, we performed a proximity ligation assay (PLA), which provided evidence for close association of pS127-YAP with p73 and, moreover, revealed that pS127–YAP–p73 complexes could only be detected in RASSF1A-expressing cells (Fig. 4f and Supplementary Fig. 6b). ChIP experiments using S127A-YAP and sip73 indicated that binding of YAP to differentiation gene promoters is pS127-YAP and p73 dependent (Fig. 4g and Supplementary Fig. 6c and d). Taken together, these observations imply that pS127-YAP contributes to ESC self-renewal and differentiation in response to RASSF1A levels. In line with this, RASSF1A overexpression was accompanied by increased expression of 'first wave' mesoderm, endoderm and ectoderm-specific differentiation markers in a p73-dependent manner^15, 49^ (Fig. 4h and Supplementary Fig. 6e). We also confirmed that normal differentiating ESC require RASSF1A expression for YAP to optimally bind to Klf3, Fbxw7 and p53 promoters and promote transcription (Supplementary Fig. 6f, g). Finally, to ultimately test our model, ESC expressing RASSF1A or control plasmid were induced to differentiate towards the neural lineage by incubating in N2B27 media containing RA. RASSF1A-expressing ESC expressed higher levels of the neuronal markers PAX3 and SOX1, in a p73-dependent manner (Fig. 4i). This indicates the requirement for an intact RASSF1A–YAP–p73 axis during ESC differentiation.

### RASSF1A regulates early embryonic differentiation in vivo

**Fig. 5:**
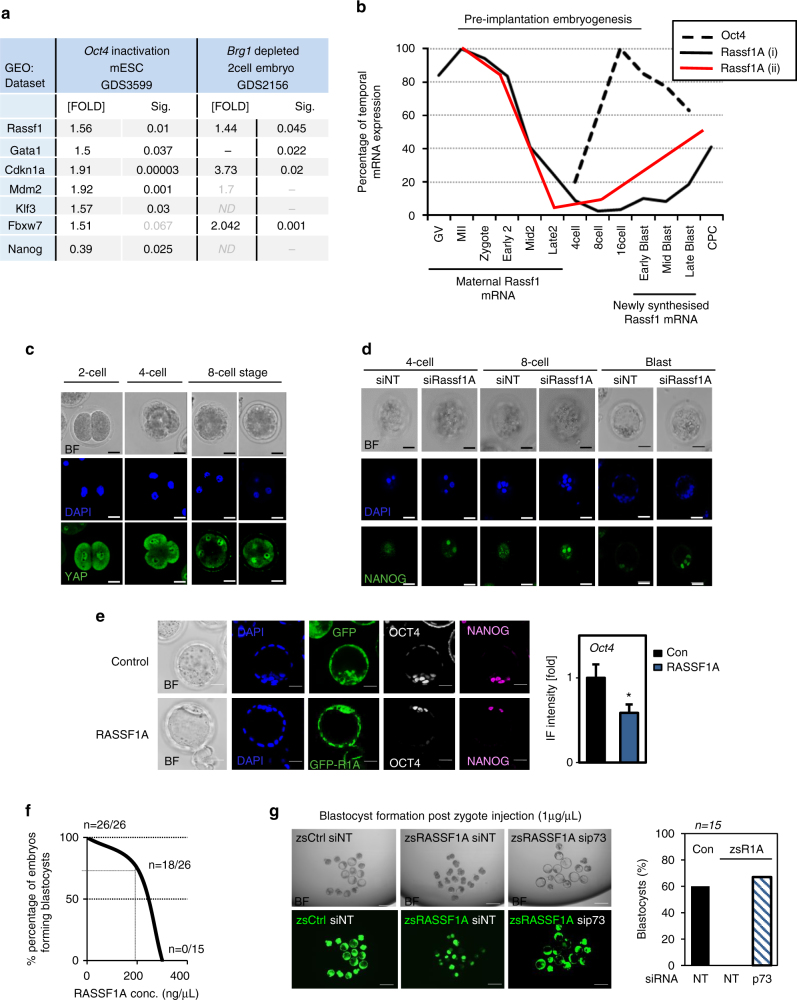
Premature activation of RASSF1A impairs embryogenesis via p73. **a** Indicated gene expression levels in published GEO data sets GDS3599 and GDS2156. **b** Temporal expression of *Oct4* and *Rassf1A* mRNA in the pre-implantation embryo (% of maximum expression) from published GEO data sets GDS752 (black colour) and GDS814 (red colour). **c** Nuclear localisation of YAP during early stages of pre-implantation development. **d** Nanog immunofluorescence and **e** representative images of embryos microinjected with either control (zsCtrl) or RASSF1A-expressing (zsR1A) vectors stained for stem cell marker expression. Bar graph showing total OCT4 protein levels across all embryos in zsR1A versus zsCtrl. **f** 'Kill curve' to determine lethal RASSF1A concentration in pre-implantation embryos. The graph expresses percentage (%) of blastocyst-forming embryos at the indicated RASSF1A concentration. **g** Viability of embryos in response to RASSF1A expression and/or sip73 microinjection, *n* = 15. BF bright field channel. Scale bars: 10–50 μm. *P < 0.05, of Student’s *t*-test. Error bars indicate s.e.m

In support of our model, expression profiles indicate that targeted inactivation of OCT4 in ESCs, pushing cells out of pluripotency towards differentiation, also increases expression of GATA1, RASSF1A and p53/p73 targets (e.g., *Mdm2*, *Cdkn1A*, *Fbxw7*) (Fig. 5a). In the early embryo, epigenetic control of totipotency is governed by Brg1, and when Brg1 is depleted in two-cell embryos, RASSF1A expression is increased along with associated differentiation genes (Fig. 5a and Supplementary Fig 7a). As YAP drives the early specification of cell fate in embryos, we reasoned that RASSF1A may play a role much earlier in development than previously anticipated. During early development the expression pattern of *Oct4* is reciprocal to *Rassf1A* indicating that our observations in cultured mESCs may be directly relevant in vivo^50, 51^ (Fig. 5b). Nuclear localisation of YAP during 2–8 cell stages of the embryo (Fig. 5c), when *Rassf1A* expression is low, is coincident with *Oct4* transcription, which in turn triggers the expression of additional core stem-cell markers (NANOG, SOX2) in the ICM^52^. To functionally test if reciprocal expression of OCT4 and RASSF1A regulates the switch to differentiation in the embryo, mouse pre-implantation embryos were microinjected with either non-targeting siRNA (siNT), siRassf1A, mRNA encoding green florescent protein (Ctrl GFP or zsGreen) or GFP-tagged Rassf1A mRNA (zsRassf1A) at the zygote stage and allowed to develop to blastocysts (Supplementary Fig. 7b). Consistently, at all developmental stages examined (2-cell, 4-cell, 8-cell, 16-cell and 32-cell stage), RASSF1A loss led to maintenance of pluripotency markers (Fig. 5d), whereas RASSF1A induction drastically reversed the phenotype (Fig. 5e), suggesting that RASSF1A can actively regulate the core pluripotency network in ESCs and the mouse embryo. Embryos injected with *Rassf1A* mRNA concentrations above 200 ng/μL did not develop and concentrations higher than 300 ng/μL led to 100% lethality (0 out of 15 embryos survived) (Fig. 5f and Supplementary Fig. 7d). Given the positive correlations between stem cell marker depletion and embryonic lethality^53^, we reasoned that the induced lethality was due to premature RASSF1A-driven embryonic differentiation. As RASSF1A appeared to exert its fate-determining functions through p73 in ESCs, we postulated that silencing of p73 should reverse the effects seen upon RASSF1A induction in the mouse embryo. To test this hypothesis, we injected embryos with 1000 ng/μL of control or *Rassf1A* mRNA, a concentration which exceeded the lethality threshold, together with siRNA to p73. Control embryos were able to form blastocysts (10 out of 15), while RASSF1A-expressing embryos arrested and died (0 out of 15 survived; Fig. 5g). Strikingly, the lethal phenotype was rescued in embryos microinjected with both *Rassf1A* mRNA and sip73, (11 out of 15 embryos survived; Fig. 5g). Knockdown of p73 alone is not accompanied by early developmental defects and embryos are viable at least up to the blastocyst stage (Supplementary Fig. 7e). Together, these data indicate that the key effector of RASSF1A signaling in development is indeed p73.

### RASSF1A represents a barrier to reprogramming in iPSC

**Fig. 6:**
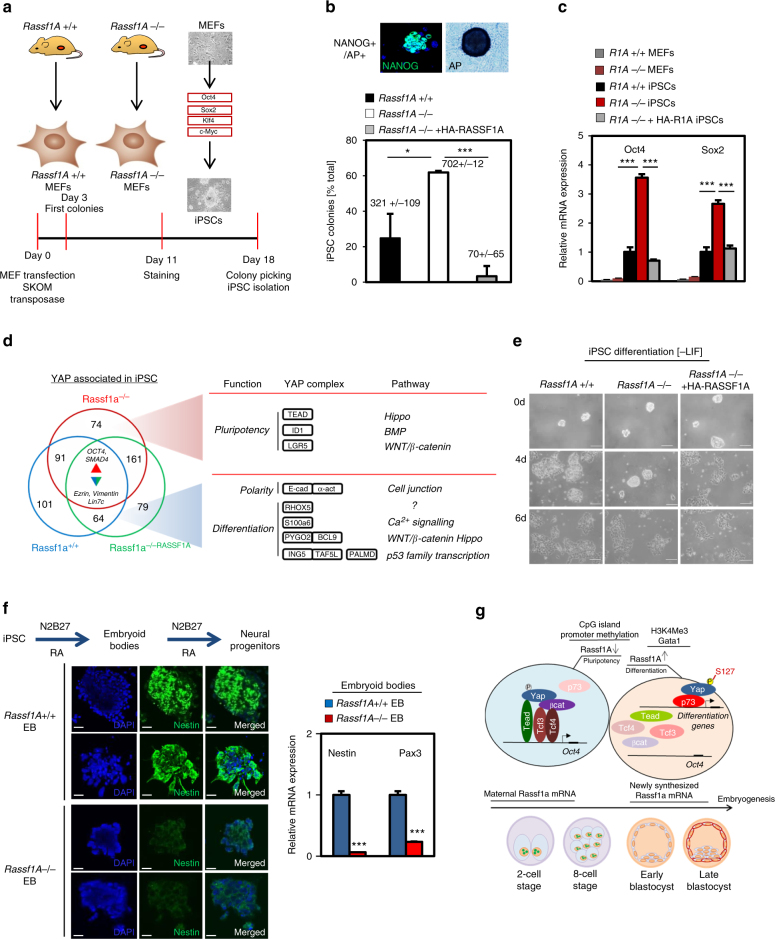
RASSF1A is a barrier to somatic cell reprogramming and iPS cell self-renewal. **a** Experimental scheme for iPSC generation from MEFs. **b** Top: example images of Nanog/alkaline phosphatase (AP)-positive round iPSC colonies and quantification of reprogramming efficiency in the respective conditions. **c** qPCR in MEFs and iPSC for core stem cell marker expression. **d** Proteomics analysis for the YAP interactome in iPSC from indicated conditions. See also Supplementary Data 5. **e** Representative iPSC colonies from indicated conditions subject to LIF withdrawal assay. Quantification is provided in Supplementary Fig 8h. **f** Neural differentiation of *Rassf1A*^+/+^ and ^−/−^ iPSC in N2B27 medium and retinoic acid (RA). Differentiation capacity of iPSC into neural progenitors is assessed via Nestin and Pax3 expression **g** Model. Scale bars: 25–50 μm. *P < 0.05, **P < 0.01 and***P < 0.001, respectively, of Student’s *t*-test. Error bars indicate s.e.m. Data shown are representative of at least three independent experiments

*Rassf1A*^−/−^ mice are viable, potentially due to compensation from the close homologue *Rassf5* (49% identity) which has similar effects in the early embryo (Supplementary Fig 7c), but tumour prone. To determine whether tumour formation was due to a failure to control the YAP pluripotency to differentiation switch we isolated mouse embryonic fibroblasts (MEFs) from *Rassf1A*^*−/−*^ and *Rassf1A*^*+/+*^ littermates and subjected them to somatic cell reprogramming as previously described^54, 55^ (Fig. 6a and Supplementary Fig. 8a). Colony morphology, NANOG and alkaline phosphatase staining indicated higher efficiency of iPSC generation from *Rassf1A*^*−/−*^ MEFs than *Rassf1A*^*+/+*^ MEF controls (Fig. 6b), suggesting that RASSF1A presents a barrier to reprogramming in agreement with results in ESCs. To confirm that the enhanced reprogramming efficiency was due to RASSF1A, we retrovirally introduced RASSF1A into *Rassf1A*^*−/−*^ MEFs (*Rassf1A*^*−/−RASSF1A*^) and found that iPSC efficiency was reduced to levels similar to *Rassf1A*^*+/+*^ MEFs (Fig. 6b).

We observed that in *Rassf1A*^*−/−*^ iPSCs, endogenous mRNA and protein levels of the core stem cell markers were elevated in comparison to *Rassf1A*^*+/+*^ or *Rassf1A*^*−/−RASSF1A*^ iPSC (Fig. 6c; Supplementary Fig. 8c). This indicates that despite equivalent expression of the reprogramming vector (Supplementary Fig. 8b), iPSC colonies were restricted from commitment to pluripotency by RASSF1A. We next wanted to confirm the regulatory switch in transcription factor association was apparent in iPSC and found reduced YAP association with p73 in *Rassf1A*^*−/−*^ iPSCs compared to control or *Rassf1A*^*−/−RASSF1A*^ iPSC and reciprocally increased association with TEAD2 and β-catenin (Supplementary Fig. 8d).

In agreement with our findings in ESCs, a proteomics screen in iPSCs identified enriched association of YAP with pluripotency associated factors in *Rassf1A*^*−/−*^ iPSCs compared to *Rassf1A*^*+/+*^ or *Rassf1A*^*−/−RASSF1A*^ iPSCs (Fig. 6d and Supplementary Data 6). pS127-YAP levels were elevated and positively associated with differentiation, promoting transcription factors in *Rassf1A*^*+/+*^ or *Rassf1A*^*−/−RASSF1A*^ compared to *Rassf1A*^*−/−*^ iPSCs (Fig. 6d, Supplementary Fig. 8e and Supplementary Data 4), thus indicating that RASSF1A is a barrier in reprogramming to pluripotency. Furthermore, following induction of differentiation via LIF withdrawal, we observed the expected increases in meso-endodermal and ectodermal markers in *Rassf1A*^*+/+*^ and *Rassf1A*^*−/−RASSF1A*^ iPSC, whereas these were lower in *Rassf1A*^*−/−*^ iPSC indicating a reduced capability to enter a differentiation programme (Supplementary Fig. 8f and g). LIF withdrawal assays demonstrated that the majority (~80%) of *Rassf1A*^*+/+*^ and *Rassf1A*^*−/−RASSF1A*^ colonies began to collapse at 4 days (80 and 95%, respectively), while 45% of *Rassf1A*^*−/−*^ colonies sustained their round shape (Fig. 6e and Supplementary Fig. 8h), suggesting delayed differentiation in keeping with higher levels of core pluripotency markers.

To assess differentiation capacity, iPSCs derived from round colonies were challenged to form embryoid bodies (EBs) and again differentiate towards the neural cell lineage, using defined media containing N2B27 and RA. Neural progenitor marker expression demonstrated that *Rassf1A*^*−/−*^ iPSCs were less efficient at generating neural progenitors (Fig. 6f), indicating a requirement for RASSF1A to promote differentiation. To further validate these results we employed a second iPSC differentiation protocol of EBs towards the dendritic cell (DC) lineage, following previously established methods^56^. FACS staining for expression of the DC-specific cell surface marker cd54 demonstrated that *Rassf1A*^*+/+*^ iPSC were more efficient at differentiating to DCs compared to *Rassf1A*^*−/−*^ iPSC, whereas both were equally responsive to LPS-mediated stimulation indicating that overall capacity to differentiate was not affected (Supplementary Fig. 8i). Taken together, these data suggest that iPSCs lacking RASSF1A exhibit general resistance to differentiation signals and maintain proliferation, illustrated by insensitivity to TGF-β-mediated inhibition and elevated Ki67 staining (Supplementary Fig. 8j and k).

## Discussion

The Hippo pathway is established to restrict pluripotency by preventing YAP–TEAD-mediated activation of *Oct4* transcription^52^. WNT/β−catenin signalling drives maintenance of pluripotency by switching the WNT effector TCF3 from a repressor to an activator of *Oct4* transcription in ESCs^57^. Here we demonstrate that these two events are mutually dependant and that both YAP–TEAD and β-catenin–TCF3 cooperate to promote transcription of *Oct4* from its distal enhancer to sustain pluripotency in ESCs. Upon differentiation of ESCs, we find that the differentiation factor GATA1 is expressed much earlier than previously appreciated and enhances expression of the MST1/2 kinase scaffold RASSF1A^37, 38^. RASSF1A drives Hippo pathway activation and pS127-YAP, which reduces YAP–TEAD interaction and transcription of *Oct4*. Of note, MST1/2 kinases are regulated by WW45/Sav1 in addition to RASSF1A, however WW45/Sav1 levels were below detection in human and mouse ESC. We find that RASSF1A-mediated Hippo activation not only restricts YAP–TEAD binding to *Oct4*, but actively promotes YAP binding to p73 to active transcriptional complexes required for ESC differentiation. Our data suggest that a TEAD-defective mutant of YAP may remain capable of supporting a p73-mediated event but this remains to be tested. Moreover, we provide evidence that the transcriptional switch is important in vivo and responsible for the recently described role for p73 in early lineage specification.

Alteration of DNA methylation is widely appreciated as a key regulatory event upon fertilisation and during induction of pluripotency in differentiated cells^58^. Under both processes, erasing epigenetic memory is key in allowing transition to distinct cellular fates. Here we find that the DNA demethylation in ESCs and pre-implantation embryos leads to reversion of the poised state of the *Rassf1A* promoter and leads to activation of *Rassf1A* transcript expression towards blastocyst formation. This is a developmentally conserved event with evidence for *Rassf1A* demethylation during zebrafish, mouse and human embryogenesis^31, 59^. In humans, RASSF1A expression has been described as one of the main clinical biomarkers of placental defects in pregnancies^33, 60^. Similarly, defects in p73 also associate with risk of recurrent pregnancy loss^61^, and our data would suggest that this may be linked to Hippo pathway signalling.

The Hippo pathway also plays a major role in the pre-implantation embryo, where YAP is required for specification of the TE and ICM^4, 32^. However, *Yap*^*−/−*^ mice do not arrest until developmental stage E8.5, implying the genetic model does not recapitulate the early developmental role^62^. Despite a crucial role in differentiation and lineage commitment, *tp73*^*−/−*^ mice only develop with late developmental defects due to compensation from p53 in the early embryo^15^. Similarly, individual genetic ablation of Hippo pathway kinases Ndr1/2, Lats1/2 and Mst1/2 only display phenotypes upon dual deletion of both isoforms, and tumour prone *Rassf1A*^*−/−*^ mice are viable, reportedly due to compensation from the homologous family member *Rassf5*^63, 64^. In keeping with the widespread role as a tumour suppressor in humans, *Rassf1A*^*−/−*^ mice succumb to a variety of tumours, indicating a potential weakening of a pluripotency to differentiation switch that is not completely compensated for in the null mice. This is supported by increased pluripotent reprogramming efficiency of *Rassf1A*^*−/−*^ MEFs suggesting that RASSF1A maintains differentiation of fibroblasts and represents a barrier to reprogramming during iPSC generation. Moreover, the loss of RASSF1A in human tumours is associated with lower differentiation grade and increased stem cell characteristics.

Taken together, our findings clearly show that RASSF1A mediates a switch of factors that YAP promotes transcription through and that this is crucial in maintaining the balance between pluripotency and differentiation. Thus, RASSF1A is a key mechanism that determines transition from pluripotency to differentiation in ESCs via WNT/Hippo pathway transcription factor competition. As RASSF1A is an established tumour suppressor, our finding of a major role in stem cell biology highlights a significant regulatory event that may contribute to tumourigenesis and provides a link between tumour formation and dedifferentiation.

## Methods

### Cell lines and culture

E14Tg2a mouse ESCs (obtained from ATCC), iPS cells and the subsequently derived stable lines (shGFP, shRassf1a, shTead2, shRassf1a/shTead2-expressing cell lines) were maintained in cell culturing medium consisting of DMEM (High Glucose, Sodium Pyruvate, Gibco) supplemented with 0.01% LIF (ESGRO, Millipore), 10% Knockout Serum Replacement (Invitrogen), 2% ES-grade Foetal Calf Serum (Invitrogen), 1% L-glutamine (Gibco), 1% non-essential amino acids (Gibco) and 0.0008% β-mercaptoethanol (Sigma). The cells were kept at 37 ^o^C in 5% CO_2_ and 20% O_2_ and cultured in gelatin-coated dishes or flasks (0.1% gelatin, Sigma). MEF cell lines were kindly provided by the David Adams laboratory, Cambridge, UK and maintained in DMEM (high glucose, sodium pyruvate, Gibco) supplemented with 10% foetal bovine serum, 1% glutamine and 1% penicillin/streptomycin at 37 ^o^C in 5% CO_2_ and 3% O_2._ Mitomycin C (Sigma, 2 mg)-treated MEF SNL76/7 cells (obtained from ATCC) were used as the feeder layer for somatic reprogramming as well as adjusting E14Tg2a cells in feeder-free culture. Cells were routinely tested and found negative for mycoplasma infections.

### Co-immunoprecipitation

For lysate preparation, 7 × 10^6^ cells were lysed in ice-cold 1% (v/v) Nonidet P40 lysis buffer (20 mM Hepes pH 7.5, 1% NP40 (Roche), 150 mM NaCl, 0.5 mM EDTA, 50 mM NaF, 10 mM β-glycerophosphate, 0.5 mM Na_3_VO_4_ and 1 × EDTA free protease inhibitors (Roche)). Lysates were cleared by centrifugation at 4 °C at 10,000×*g* for 10 min and then were subject to sonication for 15 s at 20% amplitude. Samples were normalised with Bradford protein assay and equal volumes of protein (1–2 mg) were used per reaction per experiment. Immunocomplexes were precipitated with Protein G sepharose (Sigma) or Protein G Dynabeads (Life Technologies). The antibodies used were: anti-YAP (H-125; sc-15404), anti-YAP (clone 63.7;sc-101199), anti-TEF-4 (sc-67115). IPs were incubated on an end-to-end rotator for 3 h at 4 °C and, afterwards, the beads were washed three times with NP40 wash buffer. IP samples were resuspended in 50 μL 1 × Laemmli sample buffer (12.5% glycerol (v/v), 2% SDS (w/v), 78 mM Tris pH 6.8, 720 mM β-mercaptoethanol, bromophenol blue) and boiled (100 °C; 5 min).

### Size exclusion chromatography

Whole cell extracts were prepared by resuspending frozen pellets in one packed-cell volume of buffer containing 10 mM Tris-HCl (pH 7.8), 200 mM KCl, 1 mg/ml of each protease inhibitor (pepstatin, aprotinin, chymostatin and leupeptin) and 1 mM PMSF 1 mM NEM. Five milligrams of protein in 200 μL were filtered through 0.45-μm cellulose acetate membrane and loaded onto a Superdex 200 HR 10/30 column (GE Healthcare, Little Chalfont, UK) prewashed with 2 column volumes of running buffer (20 mM HEPES pH 7.5, 150 mM NaCl, 0.5 mM EDTA, 0.25 mM DTT) and ran with the same buffer at a flow rate of 0.5 ml/min. 0.25-ml fractions were collected and 10 microlites of each fraction were analysed by Western blot. The column was calibrated with the following protein standards: thyroglobulin (669 kDa), apoferritin (443 kDa), alcohol dehydrogenase (150 kDa), bovine serum albumin (66 kDa), carbonic anhydrase (29 kDa), and aprotinin (6.5 kDa).

### Immunoblot analysis

Input samples were lysed in Laemmli Lysis Buffer (50 mM Tris-HCl pH 6.8, 2% SDS, 50 mM NaF, 10 mM β-glycerophosphate, 0.5 mM Na_3_VO_4_ and 1 × EDTA free protease inhibitors (Roche)) and normalised by NanoDrop (Thermo Scientific). Immunoprecipitates and cell lysates were western blotted as previously described^65^. The following primary antibodies were used at a concentration of 1:1000; TEF-4 (sc-67115), β-catenin (sc-7199), p73 (ep436Y), YAP (sc-15404 and sc-101199), Gapdh (abcam;2251-1; 1/10000), Tcf-3 (sc-166411), FLAG (M2; Agilent; 200472-21), Rassf1a (sc-58470), HA (Millipore;05-904), Nanog (ab80892), Oct4 (ab19857), TEF-1 (BD Laboratories;610922), Tcf-4 (Cell Signaling; C48H11/2569), LATS1 (Bethyl Laboratories; A300-477A-1), pS127 YAP (Cell Signaling; 4911), pY357 YAP (ab62751), pSer (ab9332), GATA-1 (sc-13053), MST2 (ab52641) and HRP conjugated anti-mouse and anti-rabbit secondary antibodies were used at a concentration of 1:5000 (Jackson Immunoresearch). Uncropped western blots of key experiments can be found in Supplementary Figs 9–11.

### Mass spectrometry

Samples were prepared according to the co-immunoprecipitation protocol from whole cell lysates (see above) with a few modifications. Specifically, only Protein G Dynabeads (Life Technologies) were used to precipitate the proteins of interest. Moreover, after the 3 h incubation on the end-to-end rotator, the immunoprecipitates were washed four times, the two first being with NP40 lysis buffer and the last two only with filtered PBS. Finally, the tubes were left uncapped to air-dry for approximately 15 min and no Laemmli sample buffer was added to the immunoprecipitates. The samples were subject to mass spectrometry analysis using an Orbitrap mass spectrometer.

### Methylation-specific polymerase chain reaction (PCR)

The genomic DNA from pluripotent and differentiated mouse ESC was isolated using the Dneasy Blood and Tissue kit (Qiagen) according to the manufacturer’s instructions. The methylation status of the *Rassf1a* promoter region was determined by chemical modification of genomic DNA with sodium bisulfite and methylation-specific PCR. Bisulfite treatment converts cytosine bases to uracil bases but has no effect on 5-methylcytosine bases. The bisulfite-treated DNA was used as a template for the methylation-specific PCR reaction. Primers for the unmethylated DNA-specific reaction were: FW: 5′-GGTGTTGAAGTTGTGGTTTG-3′ and REV: 5′-TATTATACCCAAAACAATACAC-3′. Primers for the methylated DNA-specific reaction were: FW: 5′-TTTTGCGGTTTCGTTCGTTC-3′ and REV: 5′-CCCGAAACGTACTACTATAAC-3′^66^.

### qPCR analysis

Quantitative Real-Time PCR was done according to the Power SYBR Green Cells-to-Ct kit protocol (Applied Biosystems). After the production of cDNA, the cDNA was diluted down 2–4 times in Nuclease-free water. The PCR Cocktail was then assembled, containing the forward and reverse primers. Reactions were performed in 96-well plates (Life Technologies) and the PCR instrument (Applied Biosystems) was programmed as follows: Stage 1. Enzyme activation (hold), repeats 1, temperature 95 °C, time 10 min, Stage 2. PCR (cycle), repeats 40, Step 1. Temperature 95 °C, time 15 s, Step 2. Temperature 60 °C, time 1 min, Stage 3. Dissociation curve, Step 1. Temperature 95 °C, time 15 s, Step 2. Temperature 60 °C, time 1 min, Step 3. Temperature 95 °C, time 30 s, Step 4. Temperature 60 °C, time 15 s. The further analysis of the results was done using the machine software. For qRT-PCR following ChIP and ChIP seq, the Input samples were at a concentration of 2.5 ng/μL. The following primers were used: *Oct4* FW: TGTGGACCTCAGGTTGGACT and REV: TTTCATGTCCTGGGACTCCTC, *Nanog* FW: AAGGATGAAGTGCAAGCGGT and REV: GGTGCTGAGCCCTTCTGAATC, *Sox2* FW: CAGGAGTTGTCAAGGCAGAGA and REV: CTTAAGCCTCGGGCTCCAAA, *Bmp4* FW: ACCAATGGAGCCATTCCGTA and REV: ACGACCATCAGCATTCGGTT, *Vimentin* FW: AGCAGTATGAAAGCGTGGCT and REV: CAGGGACTCGTTAGTGCCTTT, *Gata4* FW: GCTCCATGTCCCAGACATTC and REV: ATGCATAGCCTTGTGGGGAC, *Nestin* FW: GAGGCGCTGGAACAGAGATT and REV: CACAGCCAGCTGGAACTTTTC, *Rassf1a* FW: GTGGAGACTTCATCTGGGGC and REV: CAGCCTCATCCACGTTCGTA, *Gapdh* FW: CTCCACTCACGGCAAATTCA and REV: CGCTCCTGGAAGATGGTGAT, *Sox1* FW: CTGCAGTACAGCCCCATCTC and REV: CTTGACCAGAGATCCGAGGG, *Pax3* FW: CAAACCCAAGCAGGTGACAA and REV: TTTACTCCTCAGGATGCGGC, *Oct4* promoter (Tead2 BS) FW: GCAGAAGGTCAGGTCCACTC and REV: AGAACCCCACGACATCACTC, Oct4 promoter (β-catenin BS) FW: TGGGGGCAGAGAAGATGGTTG and REV: AAGGCAGCGACTTGGAAGCC, *p53* upstream element FW: TGGGGCTAGAAGTACCTCCC and REV: CGTGAGGGACACCATTTCCA, *Fbxw7* upstream element FW: TTAGACTGGGATGGGAGGGG and REV: GGGTTTGTGGGGGAAGAGAG, *Klf3* upstream element FW: TGGCGATTGTTGCTTGTTGG and REV: TCCCTCTCCTCTCTTCAGGC, *Tead1* upstream element FW: CCTTTTGCAGTGTTCCAGCC and REV: AGGGAAGGCTACTGAGAGGG, *Rassf1A* promoter FW: GTACAACACGCAATCCGTCA and REV: GAAGTCTCCACAGAGGTCGC, *β-actin* promoter FW: AAATGCTGCACTGTGCGGCG and REV: AGGCAACTTTCGGAACGGCG.

### Immunofluorescence

Cells plated onto gelatin-coated glass coverslips (Fisher) were fixed in 4% Paraformaldehyde solution (in PBS), permeabilised with 0.2% Triton X solution (in PBS) and blocked in 0.2% fish skin gelatin (in PBS). The cells were next incubated in primary antibody at 4 °C overnight and then washed three times with PBS before secondary antibody incubation for 1 h at RT. Cells were mounted onto microscope slides using Prolong Gold antifade reagent with DAPI (Life Technologies) and confocal microscopy was carried out using Zeiss LSM710 microscope with the Zeiss ZEN software. The following primary (dilution 1:100) and secondary (dilution 1:500) antibodies were used: Nanog (ab80892), Oct4 (ab19857), Sox2 (MAB4423), pS127 YAP (Cell Signaling; 4911), p73 (Imgenex;img-246), ki67 (AB9260), YAP (sc-15404 and sc-101199), Nestin (sc-20978), Alexa fluor goat anti-rabbit and anti-mouse (Invitrogen).

### DNA constructs and RNA interference experiments

For siRNA-mediated silencing and transient plasmid expression in ESCs, Lipofectamine 2000 (Invitrogen) was used in reverse transfections according to manufacturer’s instructions. For plasmid expression in MEFs, electroporation was performed using the Nucleofector II device (Lonza). Infections were performed with the use of HEK293T cells. shRNA sequences were cloned into the pLKO.1 puro lentiviral vector to generate stable ESC lines. Stable MEF cell lines were generated after infection with a pBabe puro retroviral vector. The following oligonucleotides were used for siRNA-mediated silencing: non-targeting (NT): 5′-TAAGGTATGAAGAGATAC-3′, Rassf1a #1 5′- CAGAACTCATTGAACTACGCGAGCT-3′, Rassf1a #2 5′-TGCGACCTCTGTGGAGACTTCATCT-3′. siRNAs to Tead2, Yap, Catnb, Trp73, Lats1, Tcf3, Tcf4, Gata1 and Tead1 were purchased from Qiagen. The following oligonucleotides were used for shRNA-mediated silencing: GFP 5′–CCGG-GCTGACCCTGAAGTTCATCTT-CTCGAG-AAGATGAACTTCAGGGTCAGC-TTTTTG-3′ Rassf1a 5′-CCGG-CTGCAAGTTCACCTGCCATTA-CTCGAG-TAATGGCAGGTGAACTTGCAG-TTTTTG-3′, Tead2 5′-CCGG-CCTCTTAGAAAGGAACCGGAA-CTCGAG-TTCCGGTTCCTTTCTAAGAGG-TTTTTG-3′. MEFs transfected with pBabe puro were cultured in the presence of 1–2 μg/mL of puromycin for 2–3 days. ESCs transfected with pLKO.1 puro were cultured in the presence of 1.75 μg/mL of puromycin for 2 days. pcDNA3 GATA1 was a gift from Licio Collavin & Giannino Del Sal (Addgene plasmid # 85693).

### Proximity ligation assay (PLA)

ESCs were transfected with the indicated constructs and 24 h post-transfection they were transferred into gelatinised (0.1% gelatin) 16-well chamber slides (Fisher) and cultured for a further 24 h. Cells were subsequently fixed and assayed using the PLA kit (Duolink) according to the manufacturer instructions. Briefly, after applying primary antibodies, cells were (1) Incubated with plus and minus probes for 1 h, (2) DNA strands were subjected to ligase treatment for 30 min, (3) DNA was amplified for 100 min with the addition of polymerase and (4) slides were mounted and PLA dots were detected via confocal microscopy (Zeiss LSM710), using the Zeiss ZEN software. The following primary antibodies were used: p73 (Imgenex; img-246; dilution 1:100) and pS127 YAP (Cell Signaling; 4911; dilution 1:100).

### Reprogramming of MEFs into iPS cells

Reprogramming of MEFs was performed by inducing expression of defined transcription factors (Sox2, Klf4, Oct4, c-Myc, referred to as SKOM)^54^. For transgene expression the piggyBac transposition system^55^ was used. SKOM transposon was used together with PBase transposase. Immediately after the transfection, MEFs were seeded on feeder layer-containing dishes and left in culture for approximately 11 days before they were stained for Nanog and alkaline phosphatase expression using the Vector Black Alkaline Phosphatase Substrate Kit II.

### Differentiation assays

For LIF withdrawal assays, ESCs and iPSCs originally cultured in complete ESC medium (ESC medium supplemented with LIF) were subject to LIF-free culture for 4–20 days and assessed periodically for morphological changes, expression of stem cell markers and differentiation capacity via microscopy, western blotting, qRT-PCR and differentiation assays. For EB formation, iPSCs were trypsinised and replated to 90 mm non-gelatinised Petri dishes (Sterilin) at a concentration of 4 × 10^5^ cells/dish in a total volume of 20 ml ESC medium in the absence of LIF. The cells were cultured for 8 days, observing periodically to ensure that the medium did not become exhausted. Proliferation of stem cells under these conditions resulted in the formation of large aggregates that comprise EBs, the latter being macroscopic at approximately day 4–5 of culture.

Differentiation towards neural progenitors was carried out as follows: EBs were allowed to be formed and then cultured in neural differentiation medium consisting of Neurobasal medium (Gibco 21103) supplemented with B27 (Gibco 17504), N2 (Invitrogen 17502-048) and RA (100 nM; Sigma R2625) for 3–4 days. Subsequently, EBs were stained for neural progenitor markers.

Differentiation towards DCs was carried out as previously described^56^. Briefly, after EB formation, all EBs were transferred to a 50 ml Falcon tube and allowed for 10–20 min to sediment under unit gravity. Following that, the medium was replaced with fresh ESC medium (LIF-free) prior to transferring to new Petri dishes. Six days later, EBs were harvested by sedimentation and washed gently twice in complete DMEM medium. The EBs were subsequently seeded at low density (approximately 10–30 EBs per plate) into non-gelatinised 10-cm tissue culture plates in LIF-free ESC medium supplemented with 2% GM–CSF-conditioned medium (Life Technologies) and 1000 U/mL of recombinant IL-3 (rIL-3;R & D systems). Following overnight culture, a proportion of the EBs attached to the dishes and began to develop colonies in a radial fashion which continued to grow until they eventually formed a stromal layer. DCs were observed around the perimeter of the colonies approximately 4 days later and continued to expand.

### Flow cytometry

Fixation and immunostaining of cells were done according to the Cell Signaling Technology protocol for Flow Cytometry. 10^6^ cells/condition were analysed on a BD FACSCalibur flow cytometer. A cd54 (I-CAM) antibody (Novus Biologicals; NBP1-72025; 1:100 dilution) was used.

### Chromatin immunoprecipitation

ChIP from ESCs was performed according to the ChIP-IT High Sensitivity kit (Active Motif). Chromatin sonication was achieved with the use of Bioruptor Plus, Diagenode, at low amplitude, pulse for 30 s on and 30 s off for 20 min in ChIP buffer. 1/20 of the chromatin preparation was extracted to generate the Input. 15–30 μg of chromatin and 4 μg of antibody per ChIP reaction were used. Binding to promoter regions was assessed via qPCR. YAP (clone 63.7; sc-101199), TEF-4 (sc-67115), β-catenin (sc-7199), GATA-1 (sc-13053), H3K4Me3 (ab8580), H3 (Cell Signalling; 14269 S), H3K27Me3 (Cell Signalling; 9733 S) and IgG (Cell Signalling; 2729 S) antibodies were used for the immunoprecipitations.

### Embryo collection and culture

Animals were maintained in the Gurdon Institute Animal Facility at 12:12 light cycle and free access to food and water was constantly ensured. Experiments were conducted in accordance with Home Office regulations. For embryo collection, 4–6 weeks old mice belonging to the C57Bl6 and CBA strains (F_1_ generation) were superovulated with 10 IU of pregnant mare serum gonadotropin (PMSG; Intervet) and 10 IU human chorionic gonadotropin (Intervet) and mated with F1 (C57Bl6 × CBA) males 48 h post treatment. Dissection of embryos out of the oviducts was performed into M2 medium and for zygote recovery cumulus cells were subjected to hyaluronidase treatment (1 mg/mL). Cultures of embryos were maintained in drops of KSOM media (Millipore) under paraffin oil in 5% CO_2_ at 37 °C.

### Embryo microinjection and analysis

Embryo microinjection was carried out on a Leica DM IRB microscope with attached Leica micromanipulators. Injections were carried out in embryos maintained in M2 medium under paraffin oil on a depression slide via air pressure with an Eppendorf Femtojet microinjector. Use of negative capacitance ensured membrane penetration.

For immunofluorescence experiments, embryos were fixed in 4% PFA for 20 min and washed 2 × 5 min in PBS-T (PBS containing 0.1% Tween).They were subsequently permeabilized with 0.5% Triton X-100 in PBS for 20 min and washed 3 × 5 min in PBS-T. The following primary antibodies were used for overnight incubation following dilution in 3% BSA (Sigma):Oct4 (Santa Cruz sc-5279; 1:200), Nanog (Abcam ab80892; 1:200) and Yap (Santa Cruz sc-15407; 1:400). Embryos were washed 3 × 5 min in PBS-T before secondary antibodies (Invitrogen, AlexaFluor) and DAPI (Invitrogen) were applied for 1 h at a dilution of 1:400 and 1:1000 respectively in 3% BSA (Sigma). Embryos were placed overnight into M2 media following 3 × 5 min washes in PBS-T and imaged on glass-bottom dishes to prevent flattening. Confocal images were acquired on a Leica SP5 microscope with a Z-resolution of 4 µm. Signal intensity differences were layer normalised using IMARIS.

zsCtrl and zsRASSF1A were cloned into pRN3p for in vitro transcription^67^. mRNA was produced using the mMESSAGE mMACHINE Kit (Life Technologies). RNA was extracted from embryos with the Arcturus PicoPure RNA Isolation Kit. qPCR was performed using SYBR Green in a StepOne Plus Real-time PCR machine (Applied Biosystems).

### ChIPseq and RNAseq data processing

ChIP seq tags were pre-processed by trimGalore (http://www.bioinformatics.babraham.ac.uk/projects/trim_galore/) to remove sequencing adaptors and low quality regions. Trimmed read alignment was performed against mouse reference genome (mouse genome version mm9) with Bowtie aligner using default parameters. We used Picard tools to remove sequencing PCR duplicates. The ChIP seq peaks were identified independently by MACS2 (http://liulab.dfci.harvard.edu/MACS/) and PePr^68^ packages. The peaks called by both methods were subjected to further analyses. Both packages were run in narrow peaks detection mode and FDR threshold was 0.01. RNA seq reads were pre-processed to remove sequencing adaptors and low quality regions as ChIP seq data. The resulting reads were aligned against reference genome by STAR aligner^69^. RNA seq raw read counts were calculated with htseq-count script using RefSeq gene annotation for mouse genome. The differential gene expression was called with DeSEQ2 R package. For integrative analysis of RNA seq and ChIP seq data, binding and expression data integration and analysis was performed by BETA software package in “plus” mode (http://cistrome.org/BETA/). The average profiles of ChIP enrichment signal within and near differentially expressed genes, as well as relative enrichment of ChIP regions in important genomic features were computed with CEAS package^70^.

Additionally, FASTQC (http://www.bioinformatics.babraham.ac.uk) quality control was performed. FASTQC provides summary statistics and quality control checks on raw sequence data. Total sequences and sequence quality scores were considered as the main criteria, with default thresholds for rejection; when residual adaptor sequences were detected they were clipped using Trimmomatic (http://www.usadellab.org). Mapping and alignment were done using Bowtie2 and TopHat2 (http://tophat.cbcb.umd.edu/). TopHat2 is a fast splice junction mapper for RNA sequencing reads; specifically, it aligns the reads to mammalian-sized genomes using the short read aligner Bowtie2, and then analyses the mapping results to identify splice junctions between exons. Paired pre- and post-treatment samples for each patient were processed using two different methods; Cuffdiff (http://cufflinks.cbcb.umd.edu) for transcript assembly and quantification of gene expression changes. Ensemble gene IDs were used as reference. Genes were ranked by their differential gene expression as estimated by Cuffdiff; rank product was used to merge samples from different conditions and to call the significantly up-regulated and down-regulated genes; The Bioconductor EdgeR package (http://www.bioconductor.org/packages/release/bioc/html/edgeR.html).

### Statistical analysis

For all experiments reported in this manuscript, at least three biological replicates were used and statistical significance was determined by Student’s *t*-test. To ensure randomization, for in vivo experiments animals were assigned randomly to either the experimental or control group. Additionally, the experiments were blinded to ensure unbiased interpretation of results.

### Data availability statement

Published GEO data sets are analysed in Figs. 1, 5 and Supplementary Fig. 7. These have the following accession codes: In Fig. 1—GSE54186, GSE3749, GDS2672; in Fig. 5—GDS3599, GDS2156, GDS752, GDS814 and GDS3599, GDS1824, GSE54186, GDS2156 were also discussed in Supplementary Fig. 7. The authors declare that all data supporting the findings of this study are available within the article and its supplementary information files or from the corresponding author upon reasonable request.

## Electronic supplementary material

Supplementary Information

Description of Additional Supplementary Files

Supplementary Movie 1

Supplementary Data 1

Supplementary Data 2

Supplementary Data 3

Supplementary Data 4

Supplementary Data 5

Supplementary Data 6
