# Supplementary material for: RASSF1A uncouples Wnt from Hippo signalling and promotes YAP mediated differentiation via p73
Source: Nat Commun. 2018 Jan 30;9:424. doi: 10.1038/s41467-017-02786-5 (PMC5789973; doi:10.1038/s41467-017-02786-5)
Supplement: Supplementary file 1 — Supplementary Information [file 41467_2017_2786_MOESM1_ESM.pdf]

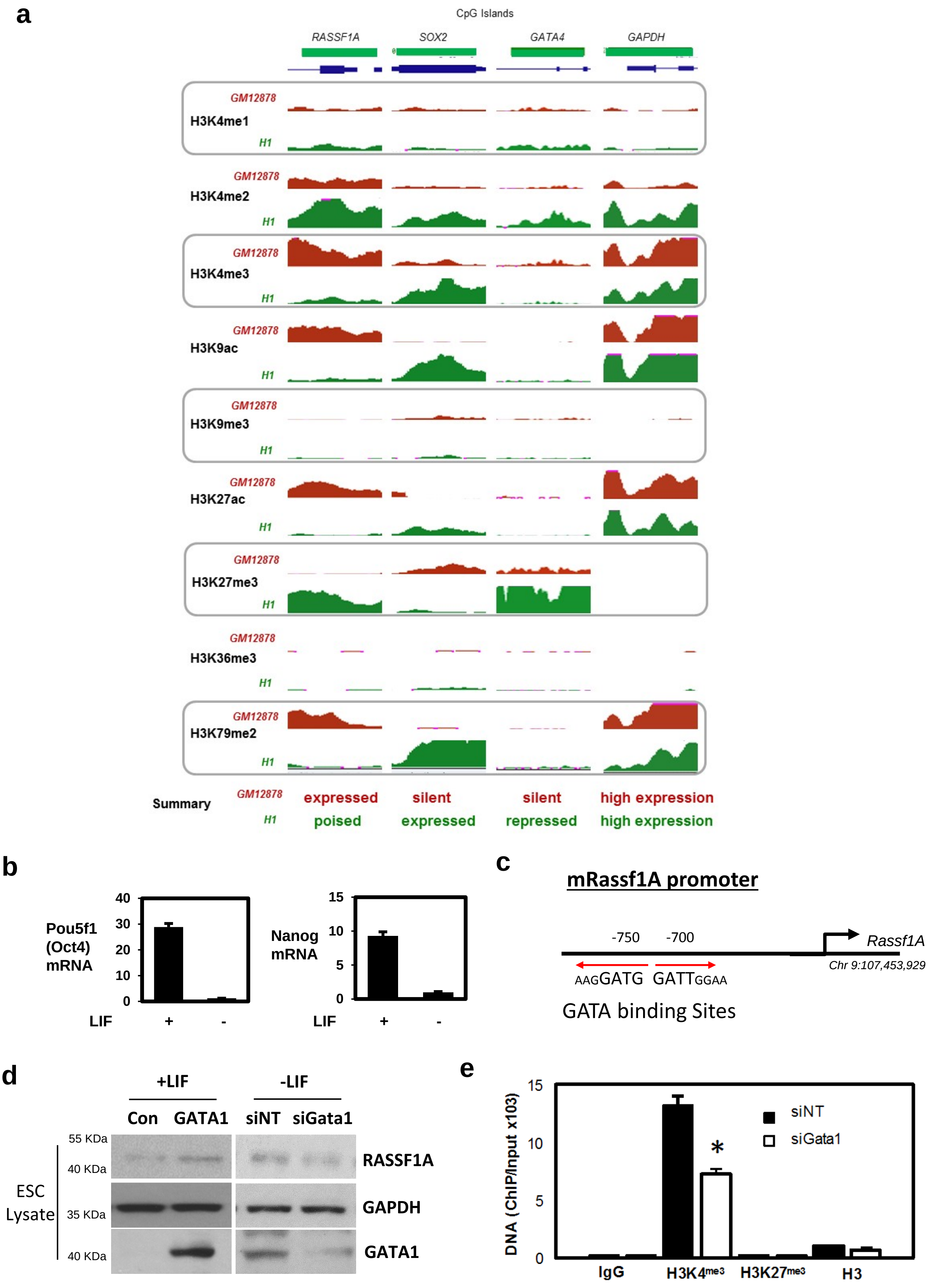
a
CpG Islands
RASSF1A
SOX2
GATA4
GAPDH
GM12878
H1
H3K4me1
H3K4me2
H3K4me3
H3K9ac
H3K9me3
H3K27ac
H3K27me3
H3K36me3
H3K79me2
Summary
GM12878 expressed silent silent high expression
H1 poised expressed repressed high expression
b
Pou5f1 (Oct4) mRNA
40
30
20
10
0
LIF + -
Nanog mRNA
15
10
5
0
LIF + -
c
mRassf1A promoter
-750
-700
Rassf1A
Chr 9:107,453,929
AAGGATG GATTGGAA
GATA binding Sites
d
+LIF
-LIF
Con GATA1 siNT siGata1
55 KDa
40 KDa
35 KDa
40 KDa
ESC Lysate
RASSF1A
GAPDH
GATA1
e
DNA (ChIP/Input x103)
15
10
5
0
siNT
siGata1
*
IgG
H3K4me3
H3K27me3
H3

Supplementary Figure 1. Epigenetic regulation of RASSF1A and GATA factor binding upon ESC differentiation (a) Histone modifications on *RASSF1A*, *SOX2*, *GATA4* and *GAPDH* promoter CpG islands in H1 embryonic stem cells versus lymphoblastoid line GM12878 representing the mesodermal cell lineage. Transcription-activating epigenetic marks (H3K4me1;H3K4me2;H3K4me3;H3K9ac;H3K27ac) are found together with repressive marks (H3K9me3;H3K27me3) on the *RASSF1A* promoter in H1 cells, indicating its function as a bivalent promoter during pluripotency, switching to its fully active state upon differentiation. The *SOX2* promoter is only active during pluripotency, whereas the *GATA4* promoter remains silent and the *GAPDH* promoter is constitutively active. The analysis was performed using data from the ENCODE Project database **(b)** Experimental controls in ESC demonstrating pluripotency marker expression in the presence and absence of LIF **(c)** GATA binding sites located upstream of the mouse *Rassf1A* promoter **(d)** Western blot demonstrating that GATA1 overexpression induces Rassf1A endogenous levels in the presence of LIF, whereas Gata1 KD reverses the phenotype even in the absence of LIF **(e)** Histone marks in siNT versus Gata1-depleted ES cells. *$*P<0.05$* of Student’s t-test. Error bars indicate s.e.m. Data shown are representative of at least 3 independent experiments.

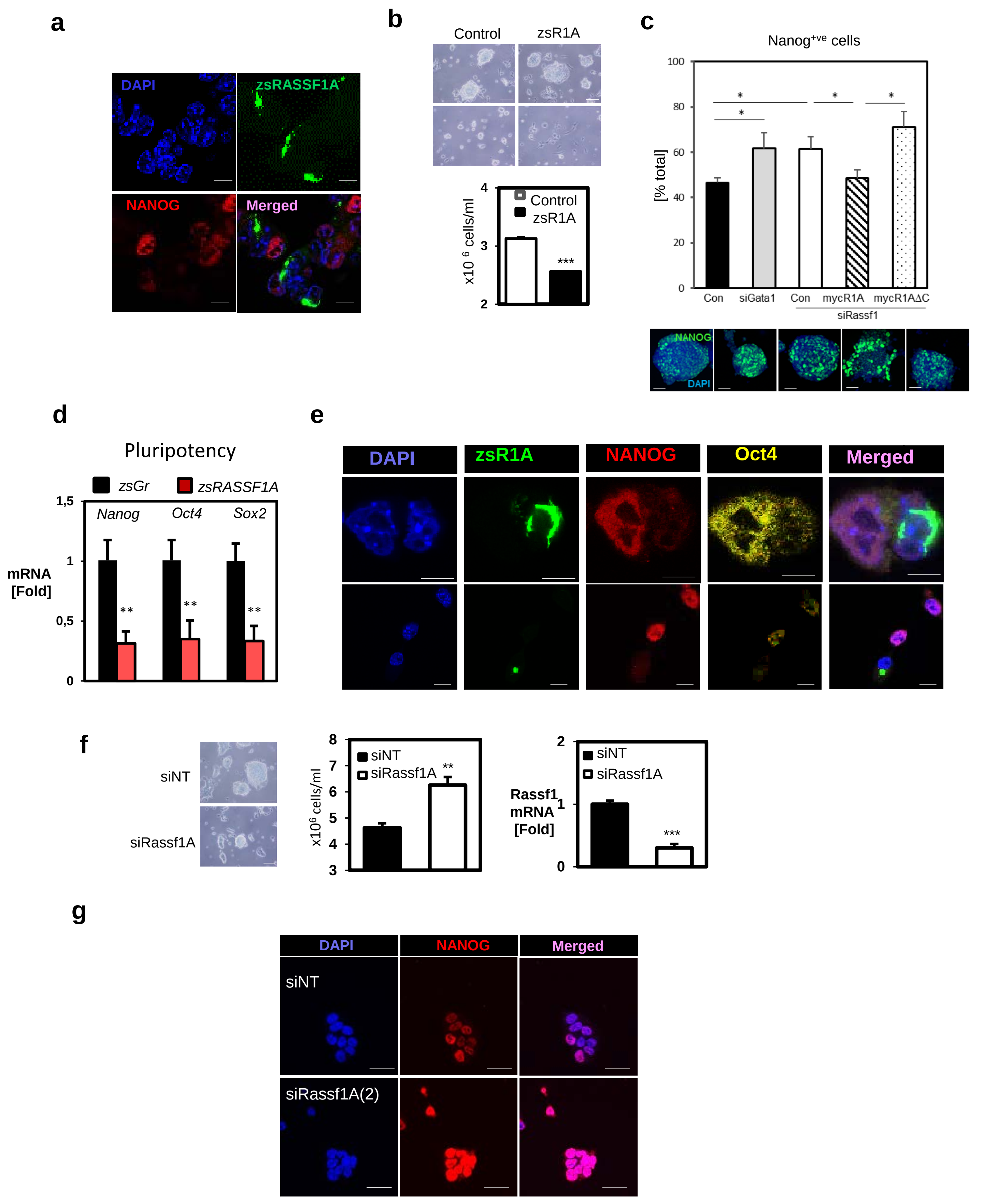
a
DAPI
zsRASSF1A
NANOG
Merged
b
Control
zsR1A
4
3
2
x10 6 cells/ml
Control
zsR1A
***
c
Nanog+ve cells
100
80
60
40
20
0
[% total]
Con
siGata1
Con
mycR1A
mycR1AΔC
siRassf1
NANOG
DAPI
d
Pluripotency
zsGr
zsRASSF1A
1,5
1
0,5
0
mRNA [Fold]
Nanog
Oct4
Sox2
**
e
DAPI
zsR1A
NANOG
Oct4
Merged
f
siNT
siRassf1A
8
7
6
5
4
3
x10^6 cells/ml
siNT
siRassf1A
**
2
1
0
Rassf1 mRNA [Fold]
siNT
siRassf1A
***
g
DAPI
NANOG
Merged
siNT
siRassf1A(2)

Supplementary Figure 2. RASSF1A regulates the ESC core pluripotency network (a) Representative fluorescent images of Nanog and zsRASSF1A (zsR1A)-expressing ESC. **(b)** Representative images and total cell numbers per condition 24 h post-transfection with the indicated constructs. Control: zsCtrl, zsR1A: zsRASSF1A **(c)** Quantification of Nanog-positive cells in the indicated conditions **(d)** RASSF1A reduces core stem cell markers at the mRNA and **(e)** protein level **(f)** Representative images and total cell numbers per condition 24 h post-transfection with control or RASSF1A-targeting siRNAs. qPCR demonstrating RASSF1A KD **(g)** Upregulation of Nanog using an alternative siRNA to Rassf1A minimises the possibility of off-target effects. Scale: 10-50 µm, $^{*}P<0.05$, $^{**}P<0.01$ and $^{***}P<0.001$, respectively, of Student's t-test. Error bars indicate s.e.m. Data shown are representative of at least 3 independent experiments.

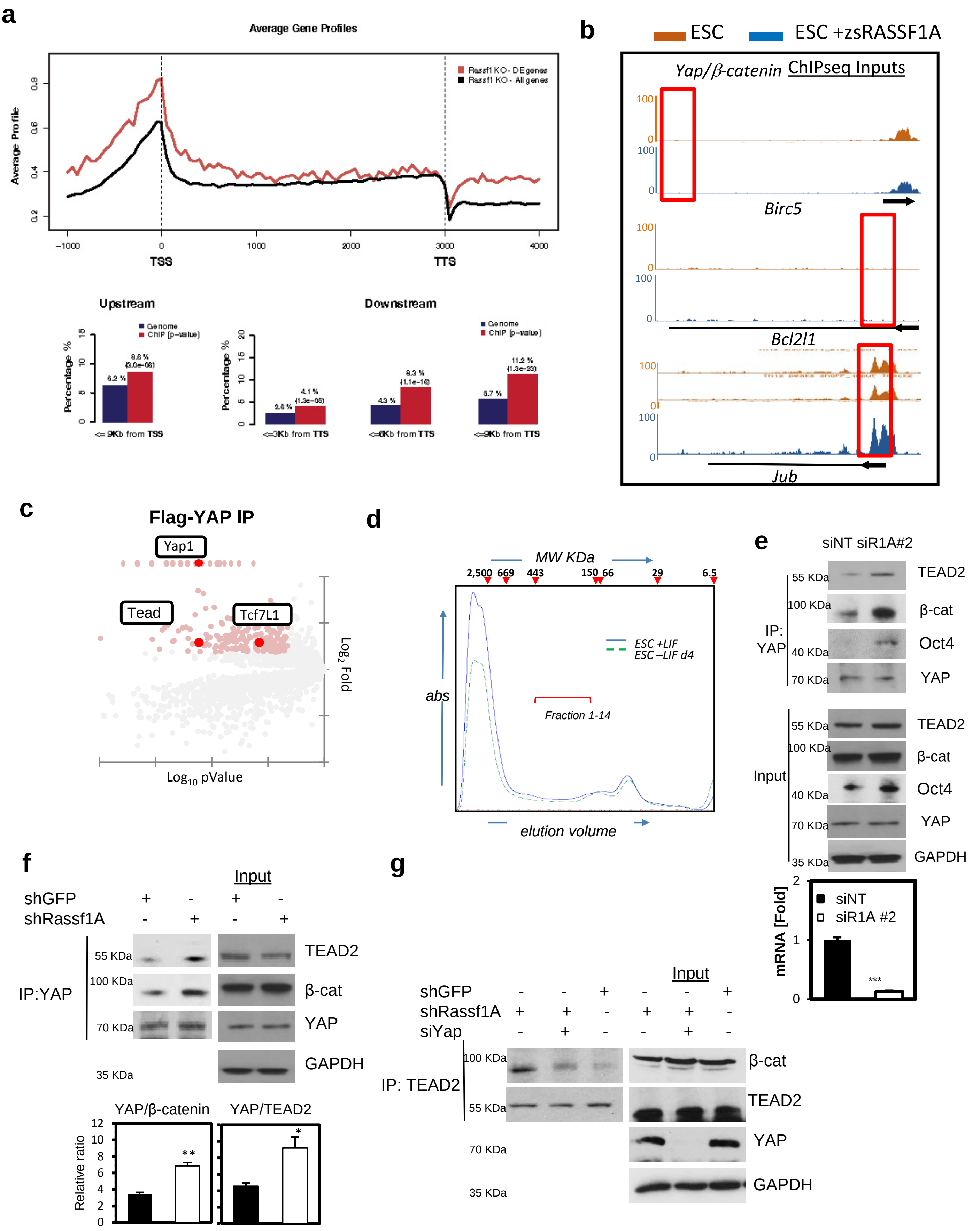
a
Average Gene Profiles
Rassf1 KO- DEgenes
Rassf1 KO- All genes
Average Profile
TSS
TTS
Upstream
Downstream
Genome
ChIP (p-value)
Percentage %
<= 9Kb from TSS
<=3Kb from TTS
<=6Kb from TTS
<=9Kb from TTS
b
ESC
ESC +zsRASSF1A
Yap/β-catenin ChIPseq Inputs
Birc5
Bcl2l1
Jub
c
Flag-YAP IP
Yap1
Tead
Tcf7L1
Log2 Fold
Log10 pValue
d
MW KDa
2,500 669 443 150 66 29 6.5
ESC +LIF
ESC –LIF d4
abs
Fraction 1-14
elution volume
e
siNT siR1A#2
IP: YAP
Input
TEAD2
β-cat
Oct4
YAP
GAPDH
mRNA [Fold]
siNT
siR1A #2
f
Input
shGFP
shRassf1A
IP:YAP
TEAD2
β-cat
YAP
GAPDH
YAP/β-catenin
YAP/TEAD2
Relative ratio
g
Input
shGFP
shRassf1A
siYap
IP: TEAD2
β-cat
TEAD2
YAP
GAPDH

Supplementary Figure 3. RASSF1A-driven YAP complexes are transcriptional regulators (a) Average profile of ChIP enrichment signal in genes differentially expressed upon RASSF1A knockdown. Relative enrichment of YAP binding sites in proximal and distal regions of the differentially expressed genes over the background (whole genome). TSS:Transcription Start Site, TTS:Transcription Termination Site **(b)** Technical controls for YAP ChIP-seq. Total sheared chromatin inputs from the indicated conditions depicting equal occupancy of classical YAP-β-catenin target genes **(c)** Volcano plot of the Flag-YAP interactome in ESC in response to RASSF1A loss. All mass-spec intensities were normalized to YAP intensities for each sample to ensure equal loading. See also Supplementary Data 3. In the absence of RASSF1A, YAP-TEAD and YAP-TCF3 complexes are stabilised **(d)** elution volumes and absorbance traces from gel filration column of ESC lysates. Fractions selected for main figure size exclusion figures indicated with red bar and elution fraction of molecular weight markers (top) **(e)** Western blotting of YAP immunoprecipitates using an additional siRNA to RASSF1A, identifying a different region of the target sequence (siRassf1A #2). qPCR showing the efficiency of siRassf1A #2 **(f)** Western blotting of Yap immunoprecipitates in shGFP and shRassf1A-expressing ESC lines, validating increased association of YAP with both TEAD2 and β-catenin upon RASSF1A loss. Bar graphs represent quantification of the TEAD2 and β-catenin relative ratio to YAP **(g)** Western blotting of TEAD2 immunoprecipitates and totals from ESC with the indicated antibodies. YAP is required for TEAD2-β-catenin complex formation. *$^*P<0.05$* and *$^{**}P<0.01$*, respectively, of Student's t-test. Error bars indicate s.e.m. Data shown are representative of at least 3 independent experiments.

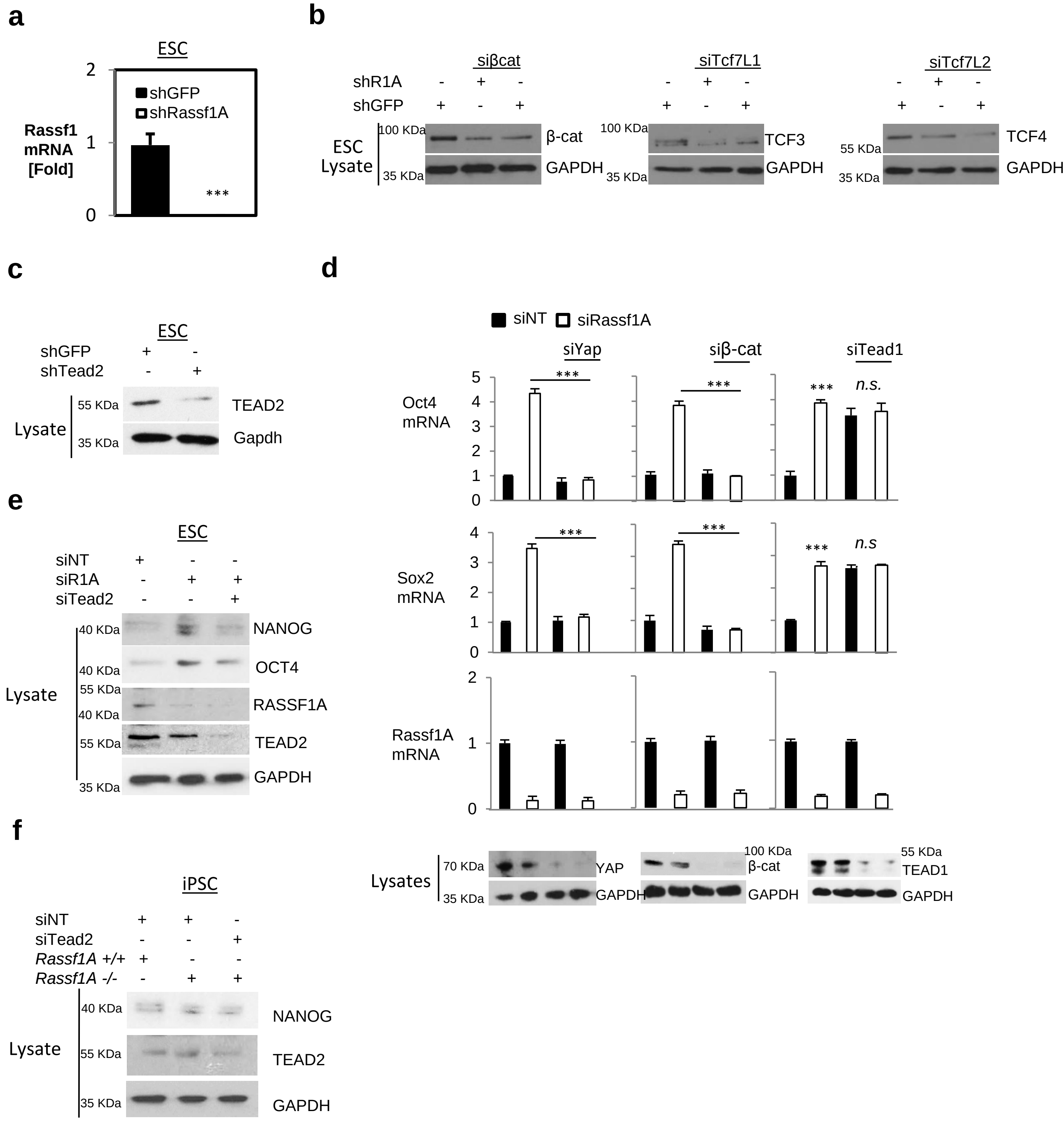
a
ESC
Rassf1 mRNA [Fold]
shGFP
shRassf1A
***
b
siβcat
siTcf7L1
siTcf7L2
shR1A
shGFP
ESC Lysate
100 KDa
35 KDa
55 KDa
β-cat
TCF3
TCF4
GAPDH
c
ESC
shGFP
shTead2
Lysate
55 KDa
35 KDa
TEAD2
Gapdh
d
siNT
siRassf1A
siYap
siβ-cat
siTead1
Oct4 mRNA
Sox2 mRNA
Rassf1A mRNA
***
n.s.
n.s
Lysates
70 KDa
35 KDa
100 KDa
55 KDa
YAP
β-cat
TEAD1
GAPDH
e
ESC
siNT
siR1A
siTead2
Lysate
40 KDa
55 KDa
35 KDa
NANOG
OCT4
RASSF1A
TEAD2
GAPDH
f
iPSC
siNT
siTead2
Rassf1A +/+
Rassf1A -/-
Lysate
40 KDa
55 KDa
35 KDa
NANOG
TEAD2
GAPDH

Supplementary Figure 4. RASSF1A loss-mediated effects require intact YAP, TEAD2 and β-catenin (a) Control for the generation of stable shRASSF1A-expressing ESC lines **(b)** Western blotting demonstrating efficient siRNA-mediated silencing of β-catenin, TCF7L1(TCF3) and TCF7L2 (TCF4) in ESC, corresponding to Fig. 3g and Supplementary Fig. 5a and b **(c)** Control for the generation of stable shTEAD2-expressing ESC cell lines **(d)** Impact of RASSF1A/YAP, RASSF1A/β-catenin and RASSF1A/TEAD1 depletion on core stem cell marker expression in ESC. Tead1 depletion is used as a control. Its depletion leads to increased stem cell marker expression, verifying its function as a differentiation marker **(e)** Impact of RASSF1A/TEAD2 depletion on stem cell marker expression in ESC and **(f)** iPSC. ****P<0.001*, of Student's t-test. Error bars indicate s.e.m. Data shown are representative of at least 3 independent experiments.

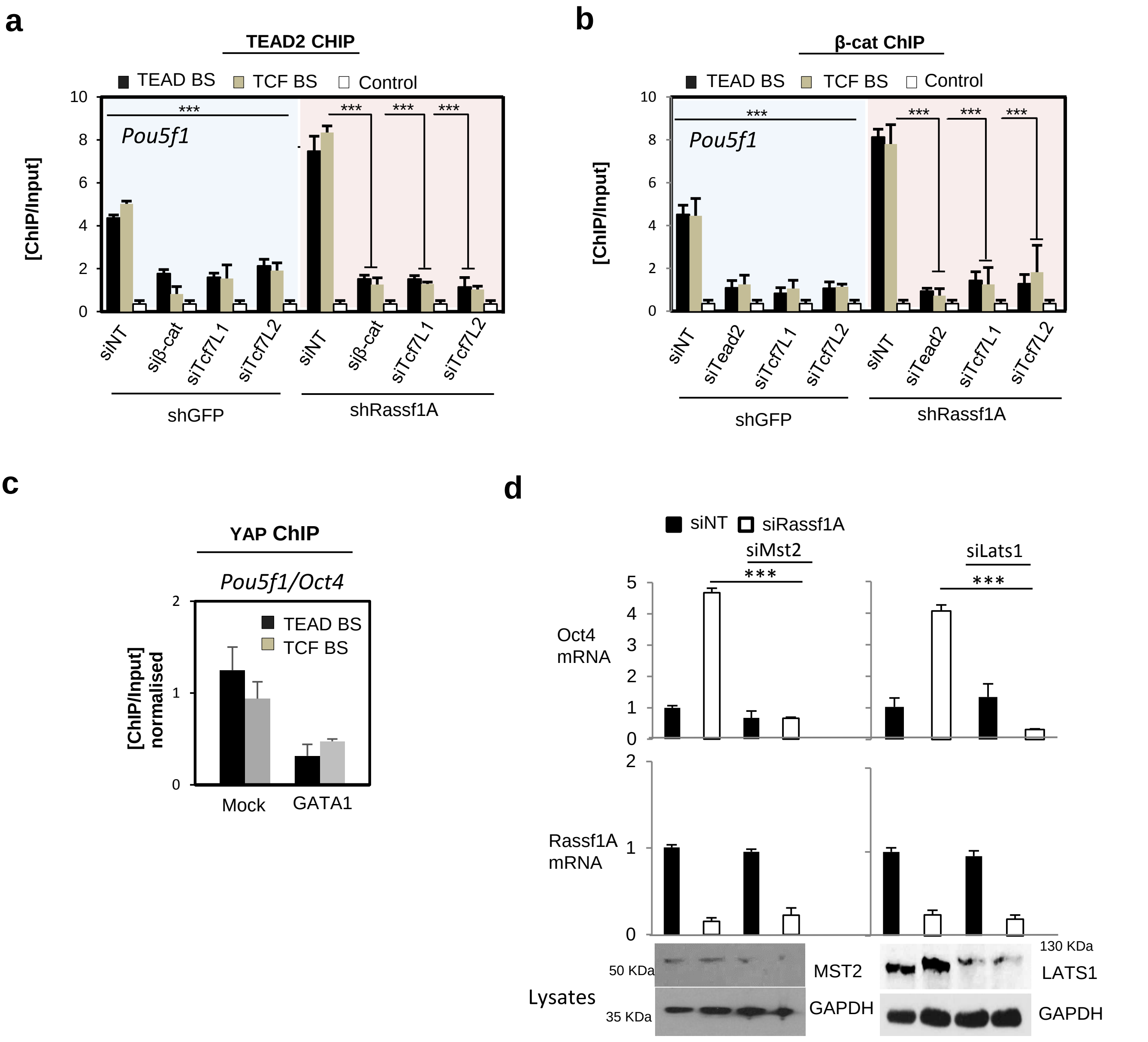
a
TEAD2 CHIP
TEAD BS
TCF BS
Control
[ChIP/Input]
10
8
6
4
2
0
***
Pou5f1
***
***
***
siNT
siβ-cat
siTcf7L1
siTcf7L2
siNT
siβ-cat
siTcf7L1
siTcf7L2
shGFP
shRassf1A
b
β-cat ChIP
TEAD BS
TCF BS
Control
[ChIP/Input]
10
8
6
4
2
0
***
Pou5f1
***
***
***
siNT
siTead2
siTcf7L1
siTcf7L2
siNT
siTead2
siTcf7L1
siTcf7L2
shGFP
shRassf1A
c
YAP ChIP
Pou5f1/Oct4
[ChIP/Input] normalised
2
1
0
TEAD BS
TCF BS
Mock
GATA1
d
siNT
siRassf1A
siMst2
siLats1
***
***
Oct4 mRNA
5
4
3
2
1
0
Rassf1A mRNA
2
1
0
50 KDa
MST2
130 KDa
LATS1
Lysates
35 KDa
GAPDH
GAPDH

Supplementary Figure 5. RASSF1A regulates the pluripotency network via the MST/LATS pathway (a) TEAD2 and **(b)** β-catenin ChIP on Tead2 and β-catenin binding sites (BS) on the ESC *Pou5f1/Oct4* promoter in response to the indicated conditions. Intact YAP-TEAD2-β-catenin transcriptional complexes are recruited on the *Pou5f1* distal enhancer upon RASSF1A loss **(c)** YAP ChIP on TEAD2 and TCF binding sites (BS) on the ESC *Pou5f1/Oct4* promoter in response to indicated conditions **(d)** Impact of RASSF1A/MST2 and RASSF1A/LATS1 depletion on core stem cell markers in ESC. The MST/LATS kinase cascade is required for stem cell marker upregulation in response to RASSF1A loss *****P<0.001*, of Student's t-test. Error bars indicate s.e.m. Data shown are representative of at least 3 independent experiments.

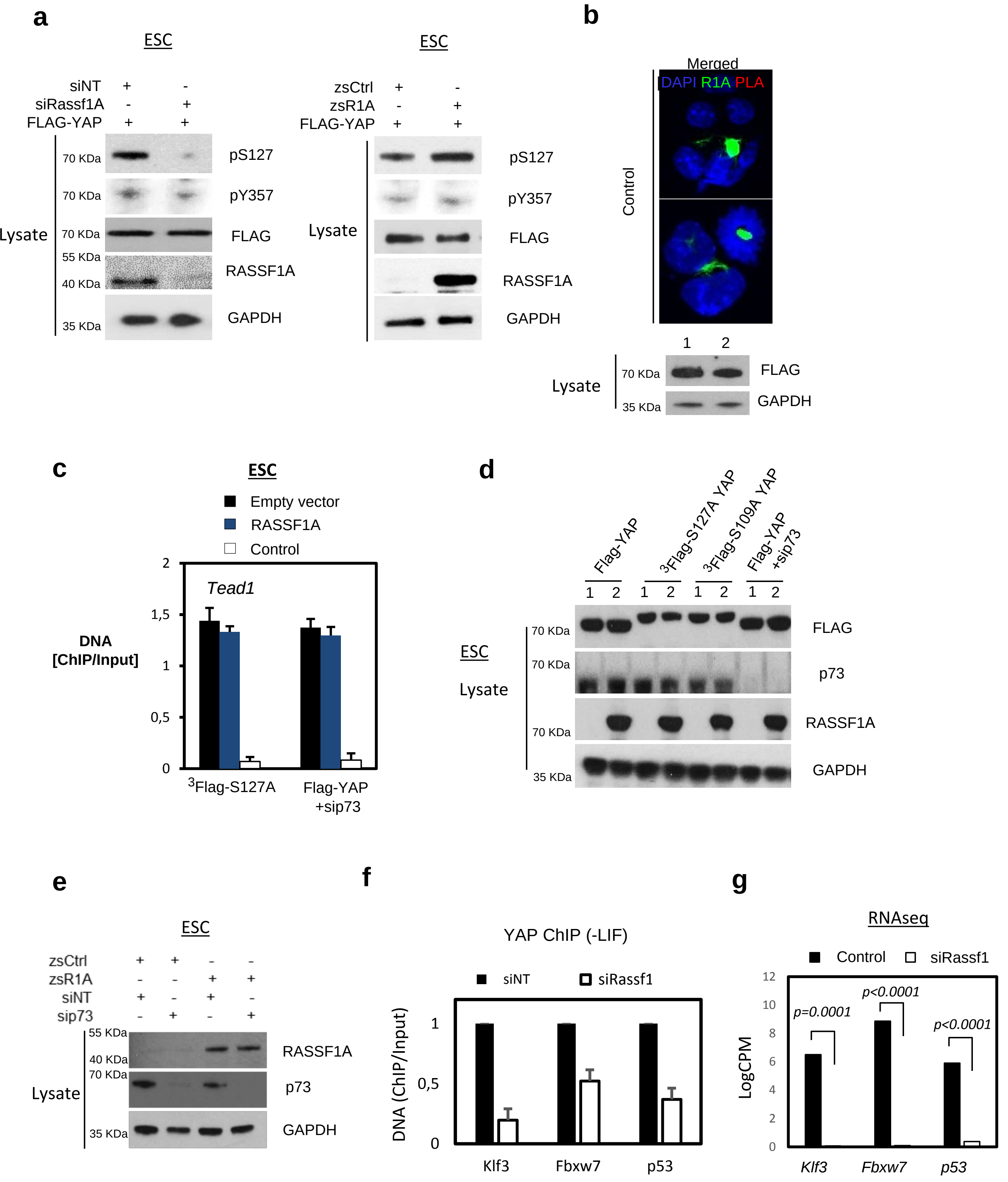
a
ESC
siNT + -
siRassf1A - +
FLAG-YAP + +
Lysate
70 KDa pS127
70 KDa pY357
70 KDa FLAG
55 KDa 40 KDa RASSF1A
35 KDa GAPDH
ESC
zsCtrl + -
zsR1A - +
FLAG-YAP + +
Lysate
pS127
pY357
FLAG
RASSF1A
GAPDH
b
Merged
DAPI R1A PLA
Control
1 2
Lysate
70 KDa FLAG
35 KDa GAPDH
c
ESC
Empty vector
RASSF1A
Control
Tead1
DNA [ChIP/Input]
2
1,5
1
0,5
0
3Flag-S127A
Flag-YAP +sip73
d
Flag-YAP
3Flag-S127A YAP
3Flag-S109A YAP
Flag-YAP +sip73
1 2 1 2 1 2 1 2
ESC
Lysate
70 KDa FLAG
70 KDa p73
70 KDa RASSF1A
35 KDa GAPDH
e
ESC
zsCtrl + + - -
zsR1A - - + +
siNT + - + -
sip73 - + - +
Lysate
55 KDa 40 KDa RASSF1A
70 KDa p73
35 KDa GAPDH
f
YAP ChIP (-LIF)
siNT
siRassf1
DNA (ChIP/Input)
1
0,5
0
Klf3
Fbxw7
p53
g
RNAseq
Control
siRassf1
LogCPM
12
10
8
6
4
2
0
p=0.0001
p<0.0001
p<0.0001
Klf3
Fbxw7
p53

Supplementary Figure 6. LATS-dependent YAP phosphorylation in response to RASSF1A (a) Western blots for S127-YAP and Y357-YAP phosphorylation levels. Y357-Yap phosphorylation was used as a control; although present, it does not change in response to RASSF1A. S127-YAP phosphorylation follows RASSF1A levels **(b)** Negative control for PLA experiment in Fig. 4f and FLAG expression in FLAG-YAP transfected ESC **(c)** YAP ChIP on *Tead1* gene promoter **(d)** Western blot controls for Fig 4g **(e)** Western blot controls for Fig 4h **(f)** YAP ChIP on differentiation-related gene promoters in response to indicated conditions in the absence of LIF and **(g)** RNAseq data displaying mRNA expression levels of the respective genes.

a

GEO: Dataset	OCT4 inactivation mESC GDS3599: GSE17439		OCT4 Knockdown mESC GDS1824:GSE4189		H1/H9 differentiation hESC GDS5408: GSE54186			Brg1 depleted 2cell embryo GDS2156:GSE5371	
Transcript	[FOLD]	Sig.	Transformed count	Sig	[FOLD] H1	H9	Sig.	[FOLD]	Sig.
Rassf1	1.56	***0.01***	UP	***0.000002***	1.23	1.49	***0.037***	1.44	***0.045***
Gata1	1.5	***0.037***	UP	***0.035***	1.52	2.92	***0.034***	-	***0.022***
Cdkn1a	1.91	***0.00003***	UP	***0.000002***	1.84	1.25	***0.015***	3.73	***0.02***
Mdm2	1.92	***0.001***	UP	*ns*		1.73	-	1.7	-
Klf3	1.57	***0.03***	UP	***0.00004***	1.93	1.32	***0.0009***	*ND*	-
Fbxw7	1.51	0.067	UP	***0.01***	1.21		-	2.042	***0.001***
Pou5F1	0.87	-	-	-	0.38	0.47	0.0000006	0.98	-
Nanog	0.39	***0.025***	-	-	0.51	0.57	0.000008	*ND*	-

b

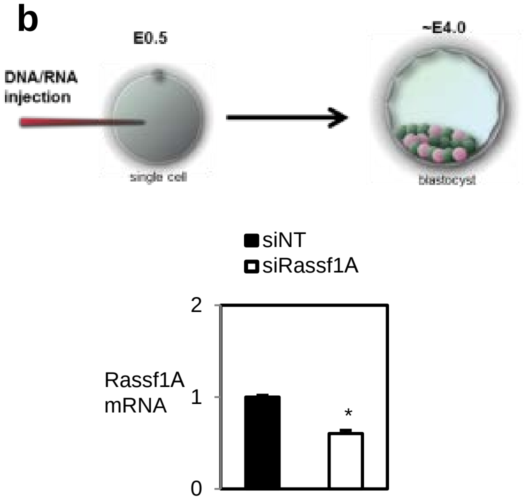

c

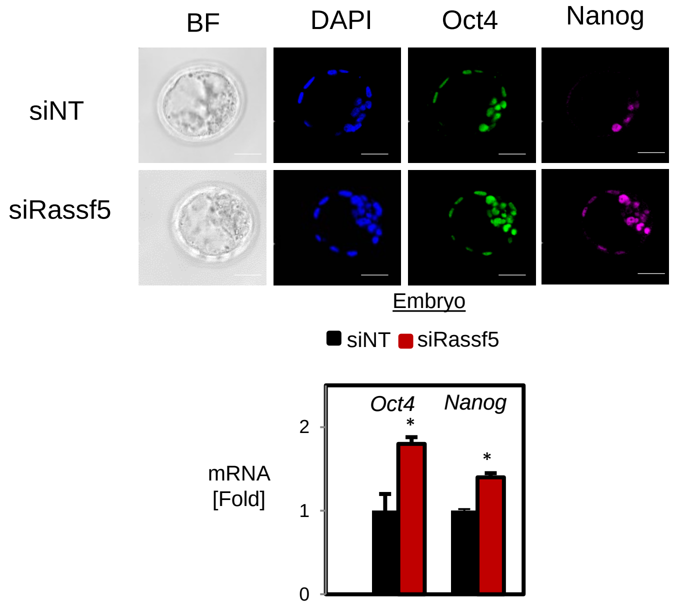

d

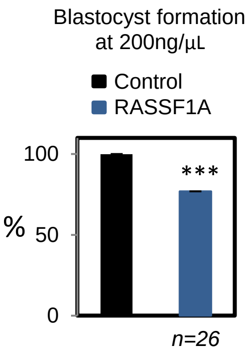

e

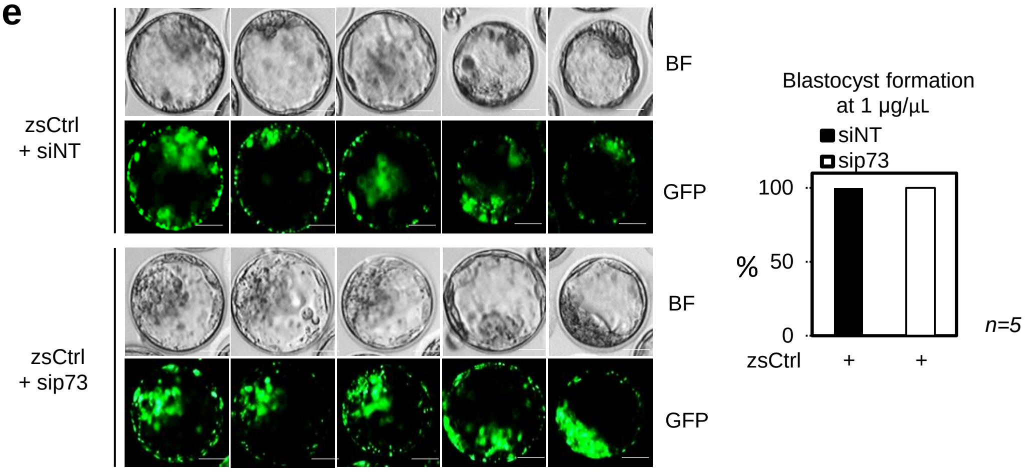

Supplementary Figure 7. RASSF1A regulates the core pluripotency network in the pre-implantation embryo (a) *Oct4* inactivation and knockdown in mESC increases RASSF1 and differentiation-promoting, RASSF1-dependent transcripts (GEO datasets GDS3599:GSE17439 and GDS1824:GSE4189 respectively). Those phenotypes are recapitulated upon differentiation of hESC lines H1 and H9 (GEO dataset GDS5408:GSE54186). Similarly, loss of the RASSF1A suppressor *Brg1* in the 2-cell pre-implantation embryo leads to increase of *Rassf1A* and Rassf1-regulated transcripts. Data deriving from GEO dataset GDS2156:GSE5371 **(b)** Top: Experimental process for *in vivo* experiments. Zygotes (E0.5) were microinjected with various fate determinants and controls and allowed to develop until blastocysts were formed (app. E4.0). Bottom: Experimental control for siRNA-mediated Rassf1a KD in the mouse embryo **(c)** Top: Representative images of embryos microinjected with either non-targeting siRNA (siNT) or siRNAs targeting RASSF5, demonstrating increased core stem cell marker expression upon RASSF5 loss. Bottom: qPCR for Oct4 and Nanog expression from the transfected embryos **(d)** Percentage of blastocyst forming embryos upon microinjection of the indicated concentration of *Rassf1A* mRNA versus Control **(e)** Control embryos microinjected with empty vector and non-targeting siRNA (siNT) or sip73 develop normally to the blastocyst stage, n=5. BF: Brightfield channel. Scale: 50 µm, $*P<0.05$ and $***P<0.001$, respectively, of Student's t-test. Error bars indicate s.e.m. Data shown are representative of at least 3 independent experiments.

Supplementary Figure 8

Papaspyropoulos et al

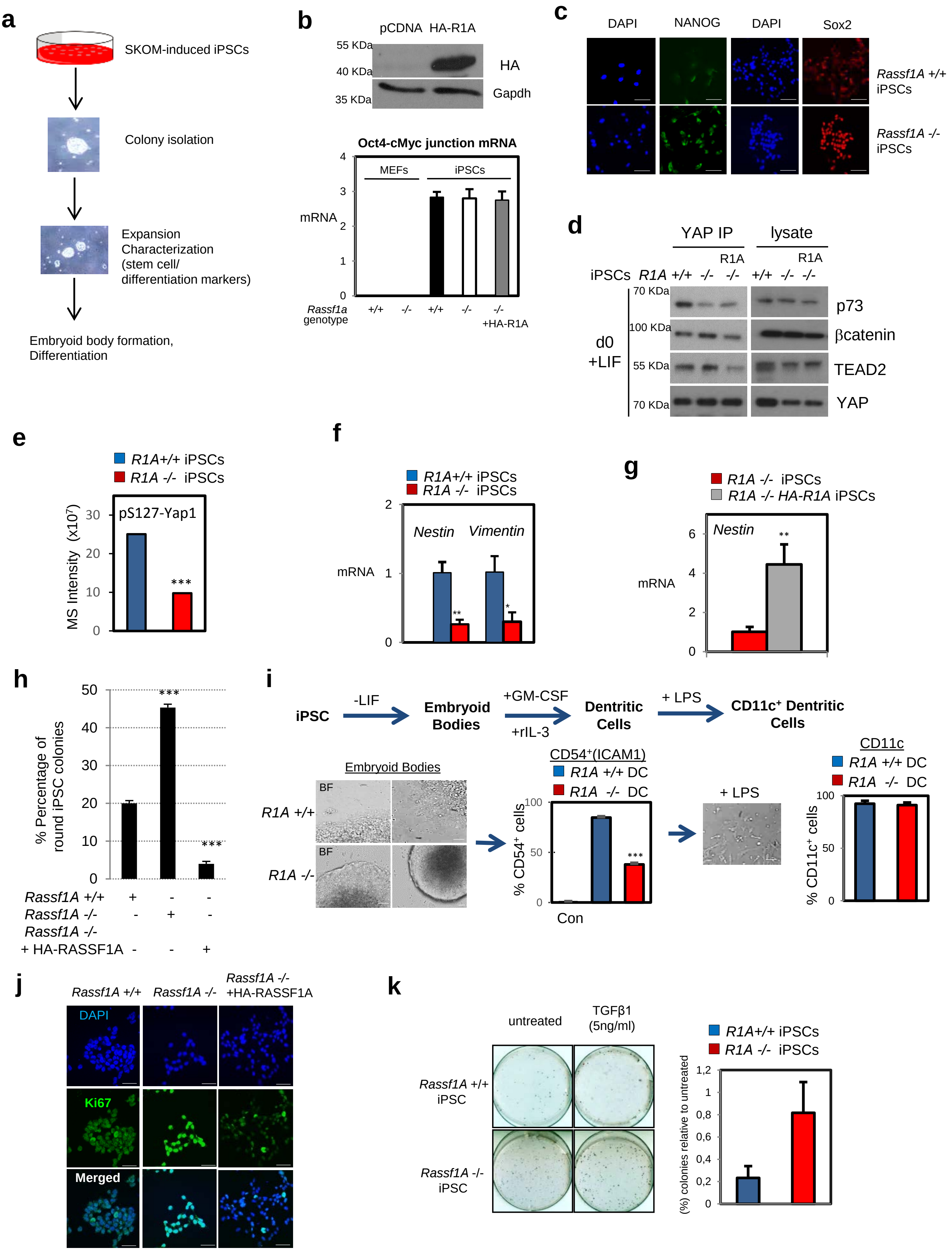

Supplementary Figure 8. Characterization of iPS cells (a) Experimental procedure of iPSC and embryoid bodies generation **(b)** Top: Western blot showing induction of HA-RASSF1A in *Rassf1A*$^{-/-}$ MEFs. Bottom: qPCR showing equal expression levels of the SKOM construct in iPSC. Untransfected MEFs were used as negative control **(c)** Representative images of indicated staining in iPSC. *Rassf1A*$^{-/-}$ iPSC express higher levels of core pluripotency markers NANOG and SOX2 than *Rassf1A*$^{+/+}$ counterparts **(d)** Western blotting of YAP immunoprecipitates in *Rassf1A*$^{+/+}$,$^{-/-}$ and $^{-/-+HA\text{-}RASSF1A}$ iPSC with the indicated antibodies **(e)** Proteomics analysis for differences in pS127-YAP in RASSF1A-expressing versus non-expressing iPSC. All cells are transfected with a FLAG-YAP1 (human) construct. All mass-spec intensities were normalized to YAP intensities for each sample. See also Supplementary Table 4 **(f)** qPCR for differentiation markers Nestin (ectoderm) and Vimentin (mesoderm-endoderm) from indicated conditions, following LIF withdrawal for 96 h **(g)** qPCR for the differentiation marker Nestin in the indicated conditions, following LIF withdrawal for 96h **(h)** Quantification of round colonies 4 days after LIF withdrawal **(i)** Embryoid bodies (EB) forced to differentiate towards the dendritic cell (DC) lineage upon GM-CSF and rIL-3 treatment. From left to right: *Rassf1A*$^{-/-}$ EBs maintain round iPSC shape; Dendritic cell (DC)-specific cd54 marker expression upon induction of differentiation, assessed via FACS. The bar graphs illustrate cd54-expressing cells in the absence of LPS; LPS used as positive control to activate DCs. The bar graphs illustrate cd11c expressing cells in the presence of LPS **(j)** Example images of iPSC staining with the proliferation marker Ki67 to assess cell cycle exit **(k)** *Rassf1A*$^{-/-}$ MEFs are insensitive to TGFβ-mediated disruption of reprogramming, in contrast to *Rassf1A*$^{+/+}$ MEFs, indicating that Rassf1A-mediated regulation of pluripotency may be dominant over induction of mesenchymal-to-epithelial transition (MET). Scale bars: 25-50 µm. $^{*}P<0.05$, $^{**}P<0.01$ and $^{***}P<0.001$, respectively, of Student's t-test. Error bars indicate s.e.m. Data shown are representative of at least 3 independent experiments.

Supplementary Figure 9

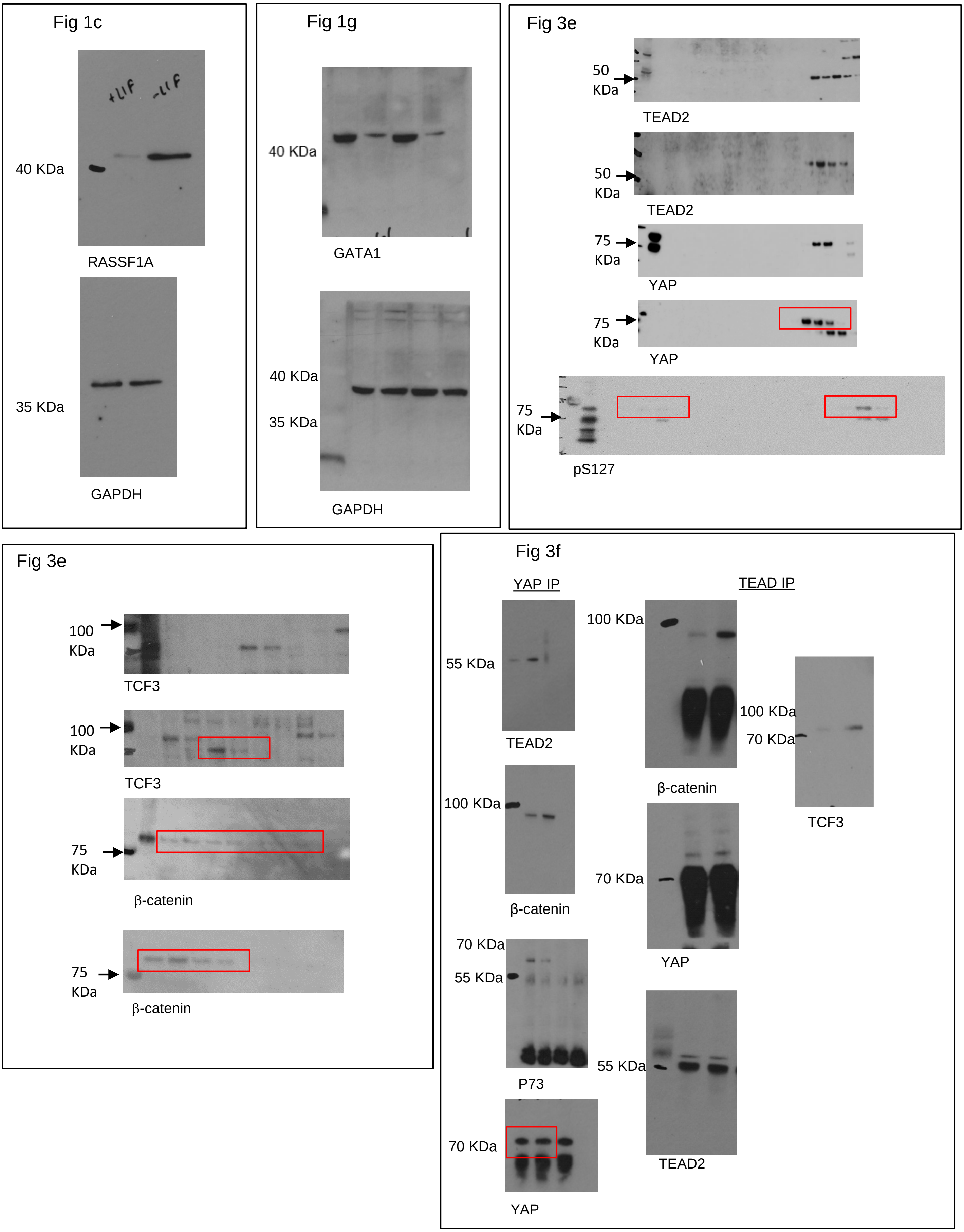

Supplementary Figure 9. Uncropped scans of blots from indicated figures

Supplementary Figure 10

Papaspyropoulos et al

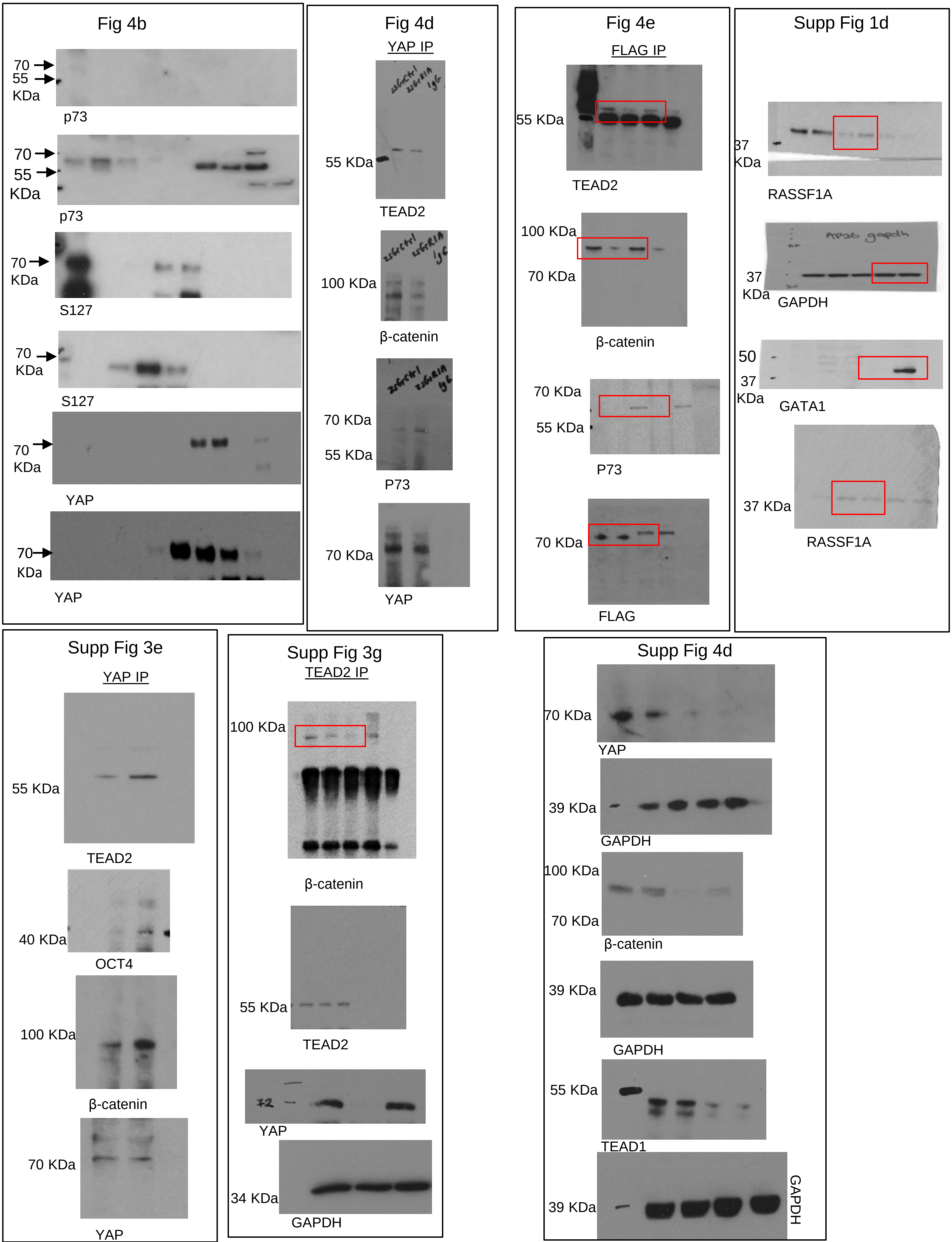

Supplementary Figure 10. Uncropped scans of blots from indicated figures

Supplementary Figure 11

Papaspyropoulos et al

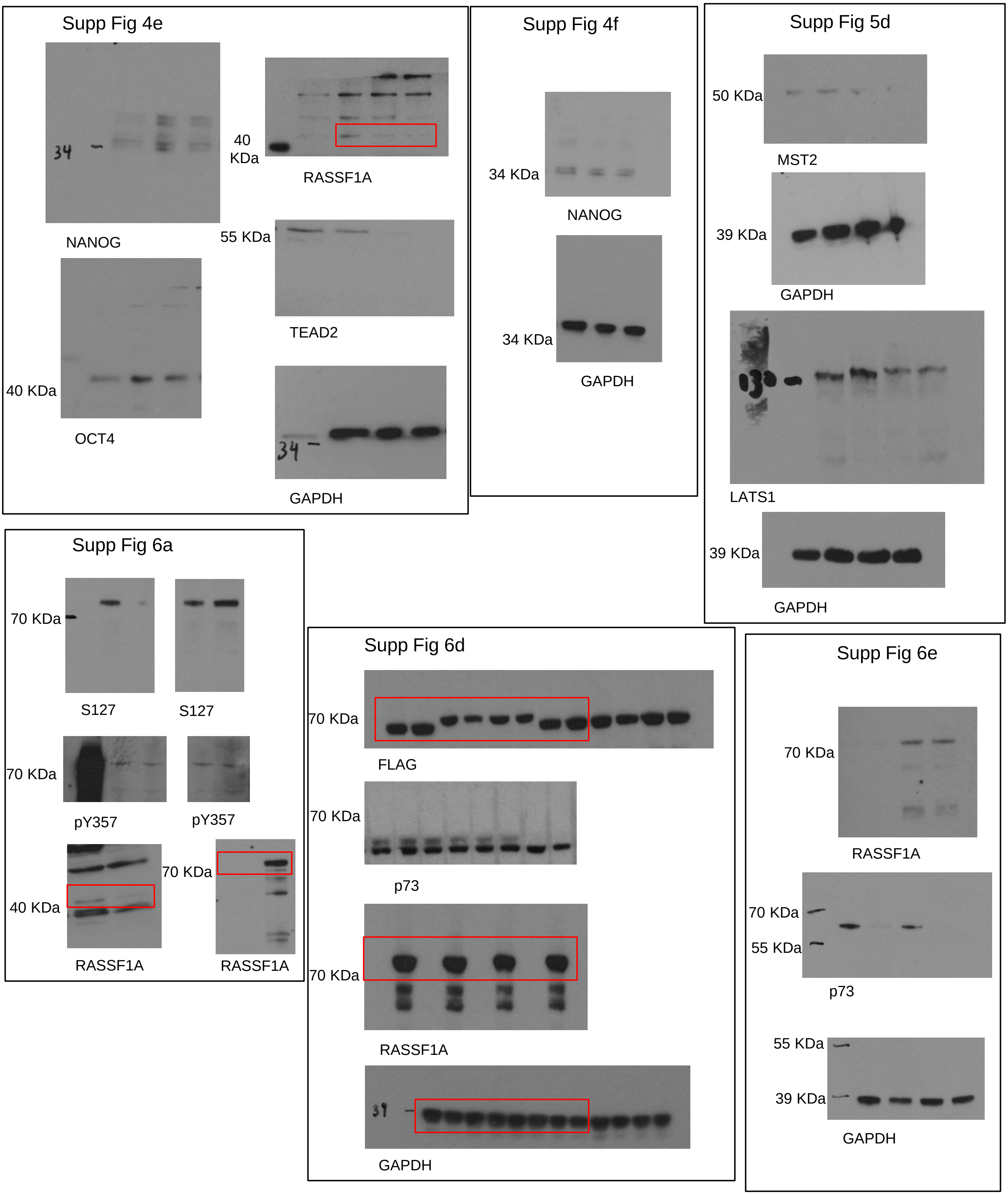

Supplementary Figure 11. Uncropped scans of blots from indicated figures
