# Supplementary material for: RASSF1A uncouples Wnt from Hippo signalling and promotes YAP mediated differentiation via p73
Source: Nat Commun. 2018 Jan 30;9:424. doi: 10.1038/s41467-017-02786-5 (PMC5789973; doi:10.1038/s41467-017-02786-5)
Supplement: Supplementary file 2 — Description of Additional Supplementary Files [file 41467_2017_2786_MOESM2_ESM.pdf]

Description of Additional Supplementary Files

File Name: Supplementary Movie 1
Description: Beating cardiac progenitor cells (CPC), resulting from mouse ESC differentiation.

File Name: Supplementary Data 1
Description: **RNAseq data.** RNAseq analysis in ESC illustrating upregulated (red) and downregulated (blue) genes in the absence of RASSF1A (shRASSF1A) versus mock (shGFP).

File Name: Supplementary Data 2
Description: **RASSF1A KD enrichment motifs**. ChIPseq data illustrating transcription factor motif enrichment in close proximity to YAP target genes in the absence of RASSF1A in ESC.

File Name: Supplementary Data 3
Description: **YAP proteomics in mESC.** Proteomics analysis for identification of YAP binding partners in mouse ESC in response to RASSF1A levels. All cells were transfected with a human FLAG-YAP construct. The first list includes peptide hits that were identified in each one of 3 individual experiments. The CRAPome database was used to discard false positive targets that are frequently found to precipitate with FLAG. Hits that displayed higher than 15% frequency on the CRAPome database and were also identified in our proteomics screen were excluded from further analysis. The second list provides the zsCtrl/zsRASSF1A ratio of LFQ intensities for each target, over a total of 3 individual experiments

File Name: Supplementary Data 4
Description: **YAP phosphorylation sites**. Mass spectrometry data depicting changes in YAP phosphorylation in response to RASSF1A levels in mESC and iPSC. All cells were transfected with a human FLAG-YAP construct.

File Name: Supplementary Data 5
Description: **YAP IP in iPSC.** Proteomics analysis for identification of YAP binding partners in *Rassf1A*$^{+/+}$, $^{-/-}$ and $^{-/-}$*RASSF1A* iPSC. Label-free quantification (LFQ) intensities were normalized to YAP intensities for each sample. All cells were transfected with human FLAG-YAP.

File Name: Supplementary Data 6
Description: **ChIPseq data.** ChIPseq analysis in mESC illustrating YAP target genes that are enriched upon RASSF1A overexpression (zsRASSF1A) versus Control (zsCtrl), as indicated by the Log Likelihood Ratio (LLR) value.
